# Disentangling sensory contributions to postural control regulation through sample entropy and neural modeling: A preliminary study

**DOI:** 10.1371/journal.pone.0355402

**Published:** 2026-08-18

**Authors:** Silvia Zanchi, Eleonora Montagnani, Victoria Marchetti, Davide Esposito, Marta Guarischi, Melissa Monti, Cristiano Cuppini, Monica Gori

**Affiliations:** 1 Italian Institute of Technology, Unit for Visually Impaired People (UVIP), Genoa, Italy; 2 Department of Electrical, Electronic, and Information Engineering Guglielmo Marconi, University of Bologna, Bologna, Italy; 3 Department of Informatics, Bioengineering, Robotics, and System Engineering (DIBRIS), Genoa, Italy; 4 Institute for Human & Machine Cognition (IHMC), Pensacola, Florida, United States of America; University of Illinois Urbana-Champaign, UNITED STATES OF AMERICA

## Abstract

Postural control relies on the integration of visual, vestibular, proprioceptive, and auditory inputs to maintain stability. While previous studies have explored the effects of individual sensory modalities, the combined influence of multisensory disruptions on postural predictability remains unclear. We preliminary assessed postural behavior during quiet standing under manipulated sensory conditions in healthy participants. Eight adults stood barefoot on a Wii Balance Board, wearing a mixed reality headset with headphones and receiving vibrotactile stimulation to the Achilles tendons. Experimental conditions combined vibrotactile, static, or moving auditory stimuli across three visual states (Eyes Open, Eyes Closed, Blurred Vision). Center of pressure (CoP) in anterior-posterior (AP) and medio-lateral (ML) planes was collected and analyzed for signal predictability using Sample Entropy. The same experimental procedures were simulated by a biologically inspired neural mass model to investigate multisensory integration in postural control at the neural level. Behaviorally, proprioceptive perturbation via Achilles tendon vibration significantly increased Sample Entropy in both spatial planes, indicating reduced system predictability, while blurred vision decreased only in the AP plane, suggesting less flexible postural dynamics. The neural model replicated key behavioral trends and also revealed distinct central mechanisms underlying sensory interactions: while auditory inputs had minimal behavioral effects, at the simulated neural level, they increased Sample Entropy, especially under dynamic conditions. This revealed subtle modulation of predictability from auditory cues, which was not detectable at the behavioral level. These preliminary results highlight the modality-specific contributions to postural control and demonstrate the utility of Sample Entropy as measure of predictability, with our computational modeling uncovering, for the first time to our knowledge, neural integration processes related to multisensory integration and postural control predictability.

## Introduction

Postural control is the ability to maintain stability against perturbations during static and dynamic conditions [1], and this depends on integration from the visual, vestibular, and proprioceptive systems [2]. Among these inputs, vision is crucial, with artificial optic flow increasing postural sway [3], while visual deprivation disrupts balance and increases fall risk [4–7]. Vision is also tightly integrated with the vestibular system, as evidenced by their shared early processing within the vestibular nuclei and the vestibulo-cerebellum [8]. With visual and vestibular cues, proprioception is also crucial for maintaining effective postural control. For example, it has been shown that numbing digital nerves with anesthesia reduces proprioceptive acuity [9]. Vibration-sensitive Pacinian corpuscles are essential for detecting high-frequency mechanical vibrations and transmitting this information to the nervous system, which allows organisms to interpret and respond to environmental stimuli [10]. Achilles tendon vibration perturbs proprioception and reduces equilibrium scores and increases the risk of falling [11–13]. Thanks to proprioceptive feedback, the central nervous system (CNS) can regulate motor programs, adapting them to environmental changes. Fundamental substrate for descending motor commands is the cerebellum, responsible to integrate proprioceptive feedback to regulate motor programs and maintain posture [14].

Contributions of the aforementioned sensory systems and their interactions in postural control are often studied with the Sensory Organization Test (SOT): this is a standardized clinical and research protocol, comprising of six conditions based on manipulations of visual and proprioceptive inputs, generally used to quantify postural control and balance by systematically manipulating the sensory inputs required for upright stance [15].

Auditory cues may also influence postural control, though findings are mixed. Studies found improved stability with spatialized sound cues, while others observe increased sway [16]. Recent research suggests that stationary broadband sounds, including white or environmental noise, may reduce the CoP sway [16,17], particularly in individuals with impaired sensory systems [18]. Traditional postural metrics include CoP displacement, sway area, and velocity. Given the nonlinear nature of biological systems, Sample Entropy can offer insights into the predictability of postural behavior under varying sensory conditions [19], with lower values reflecting more regular and predictable CoP dynamics and higher values indicating increased irregularity. However, inconsistent results have been reported across research [11,12,20,21]. Furthermore, no study has examined the effect of auditory cues or the combination of visual, auditory, and proprioceptive disruptions on postural predictability. We aimed to address this gap by preliminary evaluating postural behavior during quiet standing under manipulated sensory conditions in healthy participants. We assessed Sample Entropy in both AP and ML planes of CoP and developed a neurocomputational mass model to explain and potentially unveil new underlying neural mechanisms of postural multisensory interactions.

## Materials and methods

### Behavioral experiment

#### Participants.

Eight healthy adults (mean age (SD): 24.3 (1.3) years) with no history of neurological or sensory disorders were recruited between July 8–11^th^, 2024. The ethics committee (ASL3 Genovese) approved the study. No a priori power calculation was performed, due to the exploratory nature of the study. All participants signed informed consent under the Declaration of Helsinki.

#### Experimental setup.

**Fig 1:**
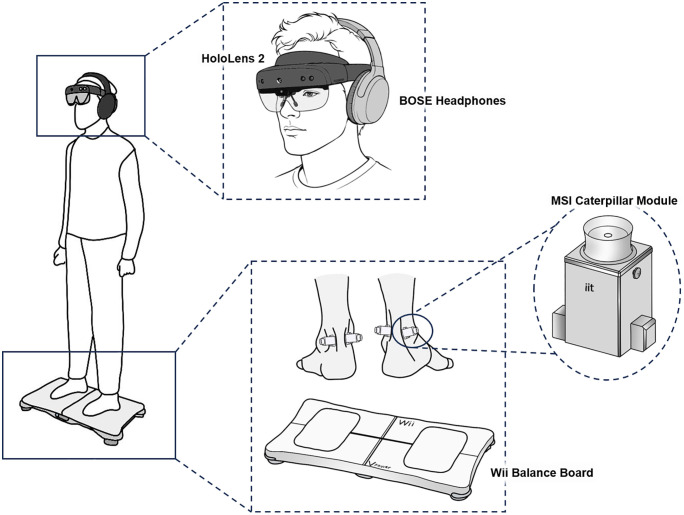
Experimental set up: Testing setup showing the participant in a relaxed stance on the Wii Balance Board wearing the HoloLens headset and BOSE headphones, with “MSI caterpillar” modules positioned medially and laterally on each Achilles tendon. The figure is not to scale.

Our setup involved an Alienware 13 R3 laptop, Wii Balance Board (Nintendo, model c; 61 × 41 × 16 cm; 113.4 g; 62 Hz), Microsoft HoloLens2 (mixed reality headset), external noise-cancelling BOSE headphones, and four customized devices called “*MSI caterpillar*” modules [22] (dimensions: 4.5x2.3x3.5 cm) (see 20) (Fig 1).

The laptop ran experimental software and processed data. The Wii Balance Board recorded CoP data [23]. The HoloLens2 was used to deliver spatialized binaural audio via BOSE headphones, while the “MSI caterpillars” delivered vibrotactile stimulation. Unity 2020 LTS was used for programming, integrating plugins: Microsoft Spatialized Audio (MRTK3;https://learn.microsoft.com/en-us/windows/mixed-reality/mrtk-unity/mrtk3overview/) for audio rendering, Holographic Remoting (MRTK3) for wireless communication, and Unity Experiment Framework [24] for experimental control. Wii Balance Board data were accessed via WiiBalanceWalker [25] and imported into Unity through a local UDP socket. CoP and head tracking data were synchronized and updated at 16 Hz.

#### Stimuli and visual states.

We used six audio-tactile conditions and their combinations. All sensory stimulations were delivered for 60 seconds. Vibrotactile stimuli were delivered at the ankle, specifically medially and laterally to the Achilles tendons [12,26], at a frequency of 180 Hz. The vibrotactile devices were strapped onto the ankle with elastic bands to ensure consistent skin contact throughout the experiment. Acoustic stimuli consisted of procedurally generated white noise at 70 dB SPL, spatialized at 1m from the participant’s head. The conditions were:

- Baseline: no sensory stimulations.
- Sound Static: two static white-noise sources were virtually positioned at 0° (front) and −90° (left) azimuth relative to participant’s head, both shifted by a + 7° angular offset to enhance spatial localization [27].
- Sound Moving: same sounds as the above, but rotating clockwise at a constant speed of 60°/s.
- Vibration: vibrotactile stimulation only.
- Sound Static + Vibration: combination of Static Sound and Vibration.
- Sound Moving + Vibration: combination of Moving Sound and Vibration.

Each condition was randomly presented across three visual states: Eyes Open, Eyes Closed, and Blurred Vision. The order of the three visual states were counterbalanced across participants. Blurring was achieved by applying an opaque nylon filter over the HoloLens display, thereby degrading the clarity of visual input without fully removing it. This manipulation was quantitatively assessed using one Early Treatment Diabetic Retinopathy Study (ETDRS) chart. The measurement was obtained at a viewing distance of 3 meters from the computer screen (1920x1080p; 23 inches) displaying the chart, using the right eye of one representative participant. The filter induced a marked reduction in visual acuity, increasing it to 0.72 logMAR (approximately 0.19 in decimal acuity), corresponding to an approximately 75% decrease in decimal acuity relative to the subject’s baseline (0.12 logMAR; ≈ 0.76 decimal acuity).

#### Procedure.

Participants stood barefoot on the Wii Balance Board, with MSI caterpillars placed on their Achilles tendons; they wore the HoloLens2, kept their arms at their sides, and looked straight ahead (Fig 1, left). Three randomized trial blocks were completed for each visual state, with each block containing six conditions. Each trial lasted one minute, totaling approximately 60 minutes of data acquisition. To prevent fatigue, 1-hour breaks were included between blocks.

#### Analysis of predictability: Sample Entropy.

Predictability is defined as how much the patterns within a time series repeat themselves throughout the sequence [19]. Standard approaches calculate time-series’ predictability using Sample Entropy. Let $\left(x_{\text{1}},\;x_{\text{2}},\ldots ,x_{N}\right)$ be a scalar time series of length N. For a given embedding dimension of m∈N and tolerance r>0, we define the embedding vectors:


$$\begin{array}{c} u_{i}^{\left(m\right)}=\left(x_{i},\;x_{i+\text{1}},\ldots ,x_{i+m-\text{1}}\right),\;i=\text{0},\ldots ,\;N-m+\text{1}. \end{array}$$

The distance between two embedded vectors $u_{i}^{\left(m\right)}$ and $u_{j}^{\left(m\right)}$ is defined as:


$$\begin{array}{c} d\left(u_{i}^{\left(m\right)},u_{j}^{\left(m\right)}\right)=\underset{\text{0}\leq k\leq m-\text{1}}{\text{max}}\left|x_{i+k}-x_{j+k}\right| \end{array}$$

Two sequences $u_{i}^{\left(m\right)}$ and $u_{j}^{\left(m\right)}$ are said to be similar within tolerance r if:


$$\begin{array}{c} d\left(u_{i}^{\left(m\right)},u_{j}^{\left(m\right)}\right)<r \end{array}$$

Let now B(m, r, N) denote the number of distinct pair (i,j) with j≠i, such that vectors $u_{i}^{\left(m\right)}$ and $u_{j}^{\left(m\right)}$ are similar within tolerance r.

Similarly, we define:


$$\begin{array}{c} u_{i}^{\left(m+\text{1}\right)}=\left(x_{i},\;x_{i+\text{1}},\ldots ,x_{i+m-\text{1}}\right), \end{array}$$

And let A(m, r, N) be the number of distinct pair (i,j) with j≠i, for which:


$$\begin{array}{c} d\left(u_{i}^{\left(m+\text{1}\right)},u_{j}^{\left(m+\text{1}\right)}\right)<r \end{array}$$

Now, the Sample Entropy of a time-series is defined as the negative natural logarithm of the conditional probability that two sequences similar for *m* points remain similar for m+1 points, within a given tolerance 𝑟, excluding self-matches [25,26], or more formally:


$$\begin{array}{c} Sample\;Entropy(m,r,N)=-\text{ln}\frac{A(m,r,N)}{B(m,r,N)} \end{array}$$

#### Raw data processing and filtering.

Data was processed in Matlab R2023b (The MathWorks, Natick, USA). Raw CoP data was captured from the Top Left, Top Right, Bottom Left and Bottom Right sensors of the Wii Balance Board. Considering its dimensions (length = 61 cm; width = 41 cm), we calculated ML and AP time series, normalizing them to participants’ body weight. This returned arrays of length N, sampled at a mean frequency of 62 Hz [23]. The time-series were filtered with a 4th-order zero-phase shift low-pass Butterworth filter, using a cutoff of 15 Hz [28].

#### Detrended fluctuation analysis.

Detrended Fluctuation Analysis (DFA) is a time-series analysis method used to quantify long-range temporal correlations in signals that may be non-stationary. This is generally performed in CoP data, which has been found to exhibit long-range correlations [29,30] that obscure time-series complexity [31]. Based on [32], we estimated self-affinity of the CoP time series using DFA, which yields a scaling exponent α that quantifies long-range temporal correlations. Values of α = 0.5 indicate uncorrelated fluctuations, whereas α ≥ 0.5 reflects persistent correlations across time scales (https://github.com/Nonlinear-Analysis-Core/NONANLibrary). AP and ML CoP time series exhibited self-affinity, consistent with long-range temporal correlations. Because the subsequent analyses assume locally uncorrelated fluctuations, we computed first differences of the time series to focus on short-term postural adjustments [29,30]. Table S1 reports α values before and after DFA computations, and Figure S1 shows an example of a log-log plot (See S1 File).

#### Input parameter selection.

We implemented a Sample Entropy function from https://github.com/Nonlinear-Analysis-Core/NONANLibrary/tree/main/matlab. Following [30], we used an empirical approach to objectively select m and r parameters for both AP and ML data. Embedding dimension (m): Using the convergence method explained in [30], we found that Sample Entropy values stabilized at *m* ≥ 3 for both CoP planes [30]. According parameters suggested by previous literature, we selected m = 4 [33]. Tolerance (r): according to [30], we estimated *r* by calculating the median across multiple calculations of the maximum relative error *Q*(*m*,*r*), evaluating Sample Entropy according to different values of r∈{0.05, 0.10, 0.15,…, 1} and values of m∈{1,…, 5} for all CoP data. We selected r = 0.25 for our analyses [33]. Reports of these sensitivity analyses are shown in Figure S2 and S3 (See S1 File).

### Neural network architecture

#### General model structure.

**Fig 2:**
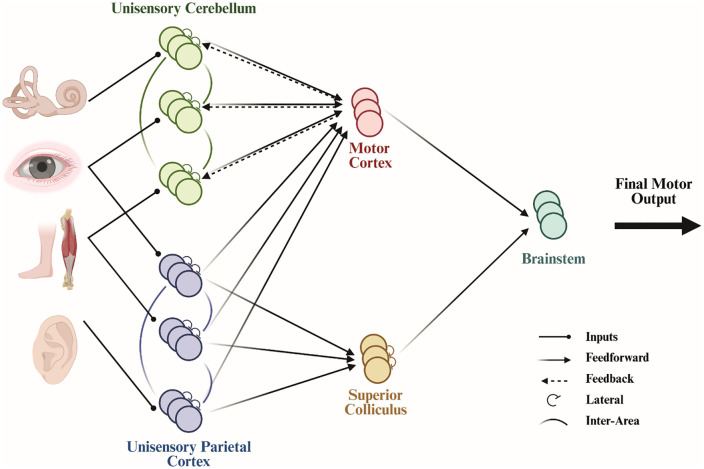
Model architecture. From the left: the vestibular, the visual, the proprioceptive, and the auditory signals reach the respective unisensory regions of the cerebellum and the parietal cortex. The inputs are processed, and this allows the superior colliculus and the motor cortex to integrate the sensory inputs and to identify the correct motor plan to preserve the vertical posture.

We implemented a biologically inspired neurocomputational mass model to identify plausible neural and synaptic mechanisms (Matlab R2022b, The MathWorks, Natick, USA). For this model, the choice of the regions involved, the connectivity and the chosen parameters were hand-tuned, based on physiologically available information [2,14,34,35]. Accordingly, we focused on key regions involved in postural regulation: cerebellum, parietal and motor cortices, superior colliculus (SC), and brainstem [14,35] (Fig 2), which transmits the final motor output. Each of these areas was designed with an equal number of neurons (N = 180), whose activation function is nonlinear and follows a sigmoid curve (Eq. S1, S1 File).

In the model, vestibular input is incorporated as a constant-amplitude sinusoidal signal (identical to the proprioceptive carrier, see Model Simulation below), representing baseline otolith and semicircular canal activity during quiet upright stance. Although vestibular input is integral to postural control, it was not manipulated across experimental conditions: all participants stood on a stable, fixed surface without galvanic vestibular stimulation or head perturbations. Consequently, vestibular drive remained approximately constant across all conditions and would not contribute differentially to condition-specific Sample Entropy differences. Indeed, including it as a dynamic, condition-varying variable would have introduced a free parameter without empirical constraint from the present dataset.

The model consists of only three layers; all layers have a known behavior, and no hidden layers were designed. Input Layer: simulating the cerebellum and parietal cortex, which process sensory cues. The cerebellum includes modality-specific modules for visual, vestibular, and proprioceptive inputs [2,36,37]. Auditory input, though processed in the cerebellum, is modeled via the parietal cortex, which integrates visual and proprioceptive signals [2,38]. These regions contribute to the body schema used for motor planning [2,34]. Integrative Layer: Includes the motor cortex and deep layers of the SC. The motor cortex selects motor plans and predicts sensory outcomes, which are compared with actual input in the cerebellum to update the body schema [2,14]. The SC integrates multisensory input to support spatial orientation and postural adjustments [2,38]. Output Layer: Outputs from the motor cortex and SC converge in the brainstem, refining motor commands before transmission to the spinal cord and peripheral effectors (Fig 2).

To simplify the model, intermediate cortical areas were omitted. Direct connections from the cerebellum and parietal cortex to the motor cortex and SC reflect their convergence in postural control pathways (Fig 2).

#### Synaptic architecture.

Information flow is organized through a structured synaptic architecture (Fig 2). Feedforward synapses (black arrows) carry sensory inputs through hierarchical layers, while feedback synapses (dotted lines) return sensory predictions from the motor cortex to the input layer, enabling comparison with incoming signals and prediction error computation (Eq. S2, S1 File). Lateral synapses within unisensory regions and the SC, along with inter-area synapses across input subregions, support local and cross-modal interactions (Eq. S3, S1 File). These connections enable multisensory integration, prediction error signaling, and body schema formation. Full mathematical details are in the S1 File.

#### Model simulation.

We generated simulated sensory inputs mirroring the experimental paradigm. The main objective of the computational model was to prove that a plausible physiological neural network involved in postural control could suggest reasonable underlying neural mechanisms exploited by the brain in such conditions. We assumed neurons in the cerebellum and parietal cortex encode vertical posture using visual, vestibular, auditory, and proprioceptive cues, each modeled according to its physiological role: *i)* Proprioceptive & vestibular inputs: Sinusoidal signals with positive values encoding sway and acceleration (oscillation frequency = 0.05 Hz); *ii)* Visual input: Constant signal reflecting a stable scene; *iii)* Auditory input: Constant in the static conditions; sinusoidal in the moving ones. These functions are kept the same for every task simulated by the network. All the connections implemented in the model are characterized by their own synaptic weight, mimicking the synaptic effectiveness, whose magnitudes were informed by the anatomical connectivity literature [2,14,34,35] and are reported in full in Table S2 (See S1 File). In addition, a uniform noise, scaled to input strength, was added to simulate sensory variability, input uncertainty, and sensory degradation. Noise-scaling parameters values were selected to reflect the ordinal severity of sensory degradation across experimental conditions. Specifically, for each sensory modality, a random term is generated within the interval [−σ, + σ], which is then scaled by multiplication with the intensity of the input signal. The symbol σ should be interpreted as the maximum amplitude of the interval defining the uniform distribution. Regarding the magnitude of the perturbation, σ defines an upper bound on the relative variation rather than an absolute quantity independent of the signal. Consequently, the signal-to-noise ratio is not constant but varies dynamically as a function of the signal amplitude itself. Within the experimental paradigm, sensory degradation was replicated by modifying the noise scaling factor, with $\sigma_{noise}$ = 1.5, while higher noise levels were used to simulate vibration ($\sigma_{noise}$ = 2) and blurred vision ($\sigma_{noise}$ = 2.5). A second noise component was added to the synaptic strength to mimic inter-individual variability in synaptic architecture. The model was tested on the experimental paradigm using the Sample Entropy of the brainstem activity barycenter (explained by Eq. S4, S1 File), computed with the same parameters of the behavior testing, for consistency. Brainstem barycenter activity reflects the spatial distribution and temporal dynamics of neural activation, and it is used as a proxy for CoP dynamics based on the established role of the brainstem in gaiting descending vestibulospinal and reticulospinal pathways. This link is supported by prior work demonstrating correlations between brainstem neural activity and peripheral CoP measures under analogous sensory manipulation paradigms [39]. Our model does not explicitly simulate spinal cord circuitry and muscle mechanics; this represents a deliberate simplification to isolate the CNS integration level from effector dynamics. We also simulated the same number of participants (N = 8) as in the experimental recordings to allow a reasonable comparison between experimental and simulated results. We further validated the neural mass model using the SOT protocol ($\sigma_{noise}$ = 1 [40]). To simulate the SOT conditions, the model inputs and the superimposed noise were adjusted accordingly. In particular, increased noise was introduced to reproduce unstable platform conditions ($\sigma_{noise}$ = 2). Tables S2, S3, and S4 of the S1 File report the values of all parameters used in the computational neural network model.

### Statistical analysis

Behavioral data were analyzed in R (v4.3.0). Trials were averaged per condition, visual state, and participants. To select the most appropriate type of ANOVA test for our data, we fitted a preliminary repeated-measures ANOVA and we assessed normality on the residuals of this first model primarily through graphical methods. In addition, we performed simulation‑based residual diagnostics using the DHARMa package, which offers a more robust assessment of normality and heteroscedasticity compared to traditional statistical tests [41]. Results from these diagnostics are presented in Figure S4, S1 File. Considering the small sample size, we opted to perform permutation ANOVA (*ezPerm* from *ez* package), a non-parametric approach widely used with small samples, where distributional assumptions are difficult to assess, and permutation methods are therefore particularly appropriate [42]. Permutation ANOVAs were run on Sample Entropy with Vibration (ON/OFF), Sound (no sound/Sound Moving/ Sound Static), and Vision (Eyes Open/Blurred Vision/Eyes Close) as within-subject factors. Participant was used as the within-subject identifier, and 10000 permutations were performed. Effect sizes were reported as Generalized eta squared (η²G [43]; eta_squared, *effectsize* [44]). Main and interaction effects were explored by performing pairwise comparisons between within-subject conditions using permutation-based paired tests (symmetry tests) implemented in the *coin* package [45]. Specifically, the function *independence_test* in the *coin* package allowed for including participant as a blocking factor, ensuring that condition labels were permuted only within each participant and preserving the repeated-measures structure of the data. For each contrast, 10000 resamples were drawn, and p-values were adjusted for multiple comparisons using the Bonferroni method. Effect sizes were reported as Cohen’s d for paired samples with Hedges’ correction applied. In addition, 95% Confidence Intervals (CI) for the paired mean differences between conditions were estimated using bias-corrected and accelerated (BCa) bootstrap resampling at the participant level, implemented using the *boot* package (function *boot*(), with BCa intervals computed via *boot.ci*()). Analyses were conducted for both AP and ML domains and applied to model simulation data.

## Results

### Behavioral experiment results

**Fig 3:**
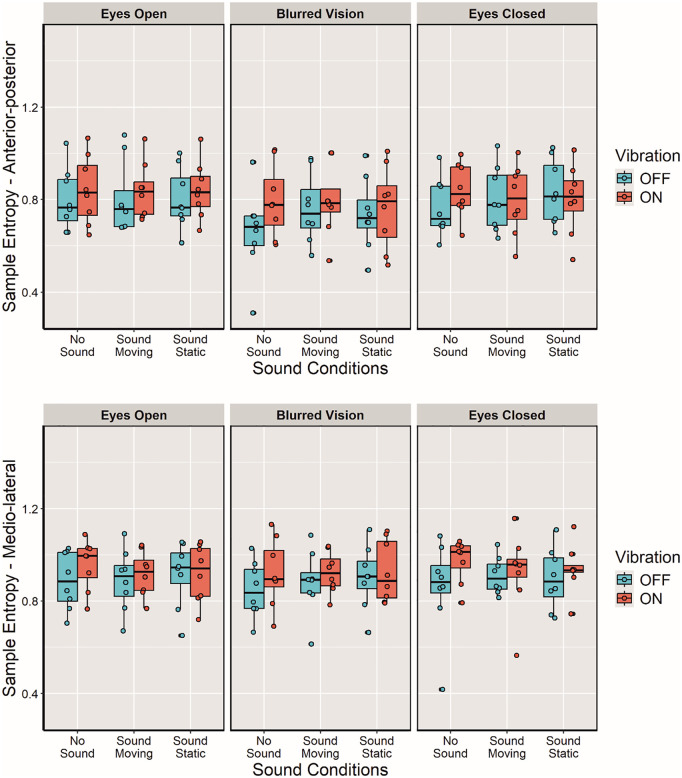
Descriptive raw data of behavioral task: Box plots illustrate median and Inter Quartile Ranges (IQR) of Sample Entropy in both the AP (upper panel) and ML (lower panel) planes, for all the sensory conditions and visual states. Whiskers extend to 1.5 × IQR. Points represent individual overlaid data.

Descriptive raw data of AP and ML can be visualized in Fig 3 (top and bottom panels, respectively).

#### Sample entropy in AP plane.

**Fig 4:**
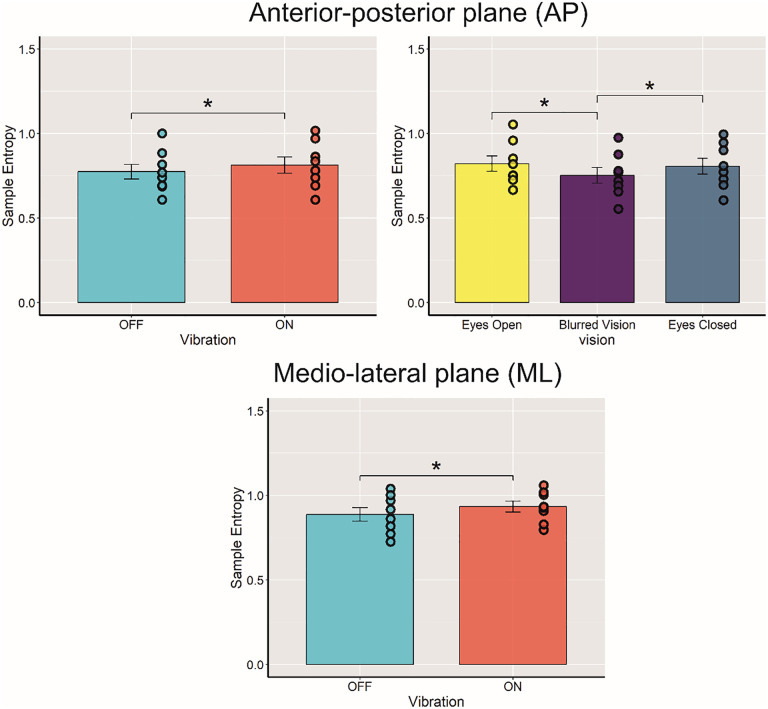
Behavioral results: Top Panel) Sample Entropy modulation in the AP plane across vibrotactile conditions and visual states: Sample Entropy increases significantly when Vibration was delivered, and it decreases significantly with Blurred Vision. Bottom Panel) Sample Entropy modulation in the ML plane. With Vibration, Sample Entropy increases. Bar plots show mean values, points represent individual observations, and error bars indicate standard error of the mean. The asterisk identifies values of p < .05.

A permutation ANOVA revealed significant effects of the visual states (*p* < .001, η²G = 0.05) and Vibration (*p* < .01, η²G = 0.02). No other effects were significant (all *p* > .08). Specifically, Vibration significantly increased entropy (Fig 4, top left panel), indicating decreased predictability of postural sway, while Blurred Vision reduced entropy (Fig 4, top right panel), indicating increased predictability of postural sway. Permutation-based pairwise comparisons showed lower entropy in Blurred Vision vs. Eyes Open (*p* = .02, mean difference = −0.07, 95% CI [−0.09, −0.05], Hedge’s g = 2.74) and Eyes Closed (*p* = .02, mean difference = 0.05, CI [0.03, 0.09], Hedge’s g = −1.38). There were no interactions between Vision and Vibration.

#### Sample entropy in ML plane.

Descriptive raw data can be visualized in Fig 3 (bottom panel). Vibration significantly increased entropy. A permutation ANOVA revealed a main effect of Vibration (*p* = .01, η²G = 0.03); no other effects were significant (all *p* > .06). Entropy was higher under Vibration than Baseline. See Fig 4 (bottom panel).

### Neural network results

#### Simulations results.

**Fig 5:**
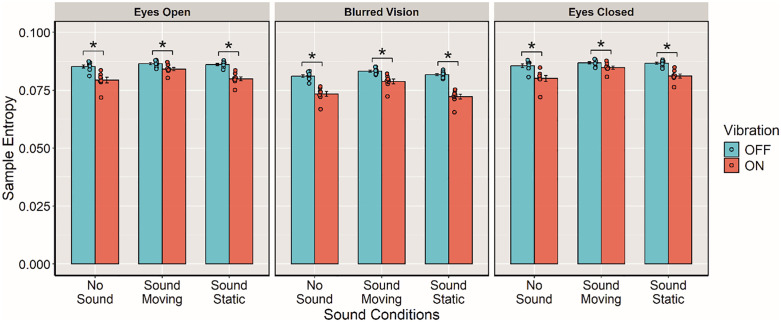
Model simulations results: Three-way interactions. Main effects: Vibration systematically led to decreased Sample Entropy, Sound Moving generally increased it, while Blurred Vision led to decreased Sample Entropy. Bar plots show mean values, points represent individual observations, and error bars indicate standard error of the mean. Details of these comparisons are reported in Table S5 of the S1 File. The asterisk identifies values of p < .05.

The neural network simulation replicated the observed trend in AP Sample Entropy under visual conditions, showing significantly lower values with Blurred Vision (Fig 5, simulation results). Unlike experimental data, Vibration reduced Sample Entropy. Contrarily, auditory inputs increased it, indicating reduced system predictability. Permutation ANOVA showed significant main effects for Vision (*p* < .0001, η²G = 0.59), Vibration (*p* < .0001, η²G = 0.62), and Sound (*p* < .0001, η²G = 0.31). These are interpreted considering the three-way significant interaction (*p* < .0001, η²G = 0.001). Generally, Blurred Vision led to decreased Sample Entropy relative to Eyes Closed and Open; Moving Sound increased entropy. In all visual states, Sound × Vibration interactions were significant (all *p* < .001, all η²G = 0.15). Specifically, for all visual states and sound conditions, Vibration systematically decreased entropy (Vibration ON vs OFF: all *p* < .026, all Hedge’s g > 1.70) (Fig 5, Simulation results; Table S5, S1 File).

#### SOT results.

We simulated the SOT paradigm [40] and applied the same non-parametric tests used for our behavioral analyses. Pairwise comparisons showed that Sample Entropy varied significantly depending on the altered sensory input, increasing with task difficulty (Table 1). The highest Sample Entropy was observed in the unstable platform condition without visual inputs. These and the interactions between Unstable Platform and Tilted Visual Surround showed moderate increases (Table 1). Such results align with Gerber et al.’s behavioral findings [40], with neural Sample Entropy in the AP plane mirroring CoP trends. Statistical analysis revealed that all conditions differ significantly, except for Baseline and Tilted Visual Surrounding conditions (Table 1).

**Table 1:** SOT results. Statistical results for pairwise comparisons across simulated SOT conditions. NV = no visual input; UP = unstable platform; TVS = tilted visual surround. P‑values were adjusted for multiple testing using the false discovery rate (FDR) correction.

Comparison	n	p adjusted	Hedges’ g	Mean difference	CI lower	CI upper
BASELINE vs NV	8	0.011	−7.6294	−0.0035	−11.5931	−3.6775
BASELINE vs UP	8	0.011	−6.1864	−0.0085	−9.4231	−2.9524
BASELINE vs UP+NV	8	0.011	−9.0872	−0.0105	−13.5729	−4.4050
BASELINE vs UP+TVS	8	0.011	−2.9569	−0.0066	−4.6074	−1.2796
BASELINE vs TVS	8	0.411	0.3179	0.0004	−0.4046	1.0193
NV vs UP	8	0.011	−3.9296	−0.0050	−6.0465	−1.7967
NV vs UP+NV	8	0.011	−5.3810	−0.0070	−8.2146	−2.5442
NV vs UP+TVS	8	0.02019	−1.6152	−0.0031	−2.6734	−0.5136
NV vs TVS	8	0.011	5.3087	0.0039	2.5074	8.1064
UP vs UP+NV	8	0.011	−2.6510	−0.0020	−4.1591	−1.1122
UP vs UP+TVS	8	0.02346	1.4277	0.0019	0.3971	2.4136
UP vs TVS	8	0.011	6.0816	0.0089	2.8995	9.2657
UP+NV vs UP+TVS	8	0.011	1.9535	0.0039	0.7162	3.1510
UP+NV vs TVS	8	0.011	6.1070	0.0109	2.9123	9.3037
UP+TVS vs TVS	8	0.011	4.5342	0.0070	2.1105	6.9477

## Discussion

In this preliminary study, we investigated how visual, proprioceptive, and auditory cues, and their interactions, shape postural control during quiet standing in healthy individuals, while maintaining constant vestibular input. To deepen the interpretation of behavioral findings, we integrated a neurocomputational mass model that accounts for observed patterns of predictability. Our model was also able to capture variations in predictability across conditions within the SOT test, a benchmark paradigm for assessing postural control, which enabled us to draw reliable conclusions about the following sensory modulations.

### Proprioception modulation

Bilateral Achilles tendon vibration increased CoP Sample Entropy in both AP and ML planes, indicating reduced predictability of the postural system. This effect likely arises from increased afferent noise introduced via Pacinian corpuscles, which are sensitive to high-frequency vibration and known to degrade proprioceptive reliability. Although our results are preliminary and based on a small sample size, they are consistent with prior studies showing that tendon vibration impairs proprioceptive acuity and posture [13]. In particular, Thompson et al. [13] reported that bilateral Achilles tendon vibration induced systematic postural adjustments, including backward body lean and increased CoP sway, reflecting a distortion of proprioceptive input used for postural control. In this context, our findings provide a complementary perspective by characterizing these effects through entropy-based measures rather than traditional kinematic metrics. Our use of Sample Entropy captures changes in the temporal structure of CoP fluctuations, suggesting that vibration not only affects the magnitude of sway but also disrupts the underlying control strategies governing postural control. Although promising, these findings should be confirmed in larger samples to establish their generalizability and robustness. Among studies employing entropy-based measures to investigate postural control changes under proprioception perturbation, one [12] reported similar increases in entropy values, while another [11] decreased entropy. This inconsistency may be attributed to methodological differences: for instance, our CoP recordings were longer (60s) compared to the shorter analysis windows used by [11] (20s), allowing for greater data capture and improved signal reliability. Furthermore, both previous studies [11,12] employed Approximate Entropy, whereas we used Sample Entropy, which is less biased by data length and more robust to noise in CoP signals [33]. Importantly, methodological differences also extend to how proprioception is perturbed. Previous studies used foam pads [20] or unstable platforms: respectively, these methods can deform consequently to different body weights hence adding inter-subject variability unrelated to the sensory manipulation itself, while unstable platforms shift the body’s reference frame and can inflate apparent entropy through multisensory conflict rather than proprioceptive degradation alone. In contrast, Achilles tendon vibration perturbs proprioception by injecting noise into the afferent stream without altering biomechanics, enabling precise assessment of sensory degradation in postural control.

Within this framework, it is important to note that Sample Entropy of the CoP increased under vibration in the behavioral task, reflecting reduced peripheral predictability, while Sample Entropy of the corresponding central neural activity decreased in the model simulations. It is important to note that the two entropy measures quantify fundamentally different dynamical systems and are not expected to vary in the same direction. Indeed, rather than reflecting a failure of the model, this divergence is consistent with a specific and testable interpretation: as proprioceptive reliability is degraded by afferent noise, the CNS may shift toward a more constrained, lower-complexity central regulatory state, producing more stereotyped central commands precisely because the incoming sensory estimate is less reliable. This is consistent with the view proposed in the nonlinear dynamics literature that motor variability reflects the structure of the underlying control process rather than simple stochastic noise [46]. Under this interpretation, the central nervous system is not failing to encode the noisy input, it is responding to that degradation by reducing the complexity of its own output, a compensatory regularization rather than a breakdown in encoding capacity. This pattern is broadly consistent with prior work reporting divergent, condition-dependent relationships between neural- and peripheral-level signal complexity during postural perturbation [39]. The specific sign and locus of the dissociation reported here represents, to our knowledge, a novel instance of this phenomenon rather than a direct replication of previously reported patterns.

The present neurocomputational model therefore reconciles the behavioral and simulated outcomes not by treating them as contradictory, but by distinguishing explicitly between peripheral sensory degradation (captured by the behavioral CoP entropy increase) and central integrative regulation (captured by the model’s central entropy decrease). This offers a mechanistic account of how a single proprioceptive perturbation can simultaneously increase noise at the periphery and decrease complexity at the integrative center responsible for postural control.

### Vision modulation

Previous studies investigating the effect of visual input on postural control using entropy measures report mixed findings, with some showing increased entropy following visual deprivation [20], others reporting reductions [30], and some detecting no changes [21]. Our preliminary analysis revealed no difference between eyes open and closed in CoP predictability. Although this result should be interpreted cautiously, given the small sample size, it is consistent with the notion that, under low-demand conditions, visual information may be effectively compensated by other sensory modalities. Discrepancies across studies may stem from methodological differences, including the choice of entropy metric, the duration and selection of analyzed time series segments, and task characteristics. For example, [20] analyzed only the central portion of the CoP signal (30s), whereas we considered the full 60s recording.

In addition, differences in task difficulty and postural constraints may influence the extent to which visual information contributes to balance control. In this context, it is reasonable to assume that our stable stance enabled sensory compensation over time, reducing reliance on visual input and minimizing its effect on CoP predictability. This interpretation can be framed within the context of multisensory integration, whereby the CNS combines different sensory signals to maintain a stable estimate of body vertical orientation [47]. When visual input is degraded, as in the blurred condition, it may still be integrated but with reduced reliability. Rather than introducing additional variability, as observed with proprioceptive modulation through vibration, visual degradation through blurring may promote a more constrained control strategy, with greater reliance on the remaining sensory channels. Consistent with this interpretation, our model predictions matched behavioral outcomes under blurred vision, unlike the divergence seen with proprioceptive perturbation. This may reflect differences in the two sensory pathways: vibration affects peripheral structures, while visual input is centrally processed and less directly linked to motor effectors. While previous work [39] showed inverse neural and peripheral signals coupling under proprioceptive disturbance, no such evidence exists for visual perturbation. Degraded visual input reduces system complexity and responsiveness, while full deprivation may be compensated for in low-demand tasks. Future works should explore dynamic visual cues (e.g., optic flow) to assess their potential to enhance postural flexibility and predictability.

### Auditory modulation

To our knowledge, no study has directly assessed the effect of auditory input on CoP predictability. Existing evidence has primarily focused on sway magnitude, with a recent systematic review reporting that auditory cues, particularly white noise, can reduce CoP sway, especially in clinical populations or individuals with impaired sensory systems [17]. While auditory cues, such as interaural time difference and interaural level difference, can support spatial awareness, they require central integration to form spatial representations. In this preliminary study, auditory inputs had no significant impact on the CoP predictability, likely due to the small sample size, task simplicity and compensatory use of visual and proprioceptive cues, which likely dominated multisensory integration processes. Even under vibration or visual deprivation, postural behavior remained stable (i.e., predictable), suggesting that it played a limited role in modulating postural control under these conditions. This is consistent with multisensory integration frameworks, where auditory cues typically play a secondary or supportive role and become more influential only when other sensory channels are unreliable or task demands increase [48].

However, our neurocomputational model revealed significant changes in the Sample Entropy of simulated central neural activity in response to auditory stimulation (i.e., sound static and moving), particularly for moving sound sources (see Fig 4, *Simulation results*). The dissociation between central and behavioral entropy outcomes suggests that auditory information may influence central spatial encoding without necessarily producing observable motor consequences in simple postural tasks. Such findings are consistent with the notion that auditory cues contribute to the building of a multimodal representation of space through higher-level integration processes [49], rather than directly driving postural adjustments. Under this account, a centrally processed auditory spatial signal can be represented with high reliability without being assigned sufficient importance by the postural control loop to produce a detectable behavioral consequence in a simple, unperturbed standing task. This interpretation is further compatible by anatomical evidence that auditory spatial processing (“where” pathway) is substantially mediated by posterior/dorsal auditory cortex and posterior parietal regions [50], which are involved in constructing internal spatial representation.

Importantly, our neurocomputational model does not take into account descending peripheral pathways that translate sensory signals into motor responses. Therefore, we have scarce information about how these effects are modulated by different systems as the body is busy maintaining optimal postural control. Nevertheless, our model helped unveil subtle mechanisms that behavioral data could not reveal, leading us to assume that dynamic auditory cues may impact CoP predictability. One possible explanation is that the neural modulation identified by the model remained below the threshold required to influence postural behavior under the relatively undemanding conditions of quiet standing. This potential effect on behavioral entropy could be revealed by developing more demanding experimental procedures (e.g., Romberg test) or controlling the use of sounds through spatial localization tasks. Moreover, further investigations could be carried out by testing elderly participants and clinical populations with sensory impairments. In addition, it is reasonable to assume that using richer acoustic environment could lead individuals to integrate sound information to modulate their postural behavior [51].

Future neural mass models should also incorporate responses of descending pathways to offer a more comprehensive understanding of how central computations influence muscle activation and balance control.

### Clinical implications

From a translational perspective, these findings may have implications for populations with impaired sensory systems, such as older adults or individuals with vestibular or neuropathic disorders. Increased CoP Sample Entropy under proprioceptive perturbation may serve as a sensitive early marker of sensory degradation that is not captured by traditional sway metrics. Furthermore, the observed dissociation between central and peripheral entropy suggests that behavioral stability may mask underlying neural instability, which could contribute to fall risk under more demanding conditions. The potential influence of auditory cues at the CNS level also opens avenues for rehabilitation strategies. For instance, enriched or spatialized auditory environments could be leveraged to enhance sensory integration in patients with visual or proprioceptive deficits. However, it is plausible to assume that such effects may only emerge under sufficiently challenging conditions, highlighting the importance of task design in clinical assessment protocols.

### Limitations and future directions

This study has few limitations. First, the sample size was relatively small, which may limit statistical power, reliability and the generalizability of the findings. Future studies with larger cohorts are needed to confirm the robustness of these results. Second, we acknowledge that a formal parameter sensitivity analysis for the neurocomputational mass model was not performed in the present study. Future work will address it by adopting biologically validated neuro-musculoskeletal models of the spinal-peripheral effector stage (such as those developed by [52]) that would also provide the physiological constraints needed to anchor parameter ranges more tightly. Third, the neurocomputational model does not incorporate descending pathways linking central processing to motor execution. As a result, it cannot fully capture how sensory integration is translated into muscle activation and postural adjustments. A principled route toward closing this gap is to couple the present central model to a biologically based spinal-peripheral effector stage, such as the large-scale neuro-musculoskeletal model of Elias et al. [52]. Coupling our central, modality-specific multisensory integration model to such a peripheral effector stage would allow the descending output of the present network to drive a physiologically grounded spinal-muscular system, closing the loop between central sensory integration and peripheral motor execution, and providing a principled account of how central Sample Entropy modulation propagates to peripheral CoP variability. We consider this integration as the most direct extension of the present work and outline it as a concrete avenue for future research. In addition, this study did not include measures of direct neural recordings (e.g., EEG/fMRI); model-derived considerations on central dynamics are therefore simulated rather than empirically observed, and the absence of neural correlates limits the ability to validate model predictions and to directly relate central dynamics to behavioral outcomes. Finally, the study did not include direct measurements of muscular activity (e.g., EMG), which would be required to validate how central sensory integration, as captured by the present model, propagates to peripheral motor output under the different sensory conditions tested.

## Conclusions

Overall, this preliminary study developed a computational framework linking central neural dynamics, quantified through entropy-based measures, to multisensory processes underlying postural control. This integrative approach provides a mechanistic account of how sensory information supports balance. Our results show that postural stability emerges from multisensory integration with modality-specific effects: Achilles tendon vibration reduces postural predictability by disrupting proprioception, while visual degradation promotes more rigid control strategies. Auditory inputs produce minimal behavioral effects under simple conditions but may modulate central dynamics not captured by CoP measures. The neurocomputational model highlights integration mechanisms not observable in behavior alone, supporting the value of combining experimental and modeling approaches. Future work should incorporate neural recordings, extend models to include descending pathways, and examine more complex and ecologically valid tasks. Studies in larger and clinical populations may further clarify the role of multisensory integration in postural stability and control.

## Supporting information

S1 File Table S1. Alpha values before and after adjustment for long-range correlation; Figure S1. Detrended Fluctuation Analysis before and after correction; Figure S2. Selection of embedding dimensions (m) for AP and ML planes of CoP; Figure S3. Selection of tolerance radius (r); Eq. S1: Mathematical definition of neurons nonlinear activation function; Eq. S2: Mathematical expression of the feedforward and feedback synapses designed based on a Gaussian distribution; Eq. S3: Mathematical formulation of the lateral and inter-area synapses following a Gaussian distribution; Eq. S4: Mathematical computation of the Sample Entropy of the barycenter of the brainstem activity; Table S2. Feedforward (from column 2–5) and feedback synapses (last column) connecting the neural network areas; Table S3. Lateral synapses (left) and inter-area synapses (right); Table S4. Input intensities in the experimental and in the SOT paradigm; Figure S4. DHARMa residual diagnostics for our model on Sample Entropy data in AP, ML and simulated data; Table S5. Statistical results from permutation‑based paired comparisons (n = 10000 permutations) performed on the simulated dataset.
(DOCX)
