# Supplementary material for: Disentangling sensory contributions to postural control regulation through sample entropy and neural modeling: A preliminary study
Source: PLoS One. 2026 Aug 18;21(8):e0355402. doi: 10.1371/journal.pone.0355402 (PMC13485045; doi:10.1371/journal.pone.0355402)
Supplement: Supplementary file 1 [file pone.0355402.s004.pdf]

Manuscript ID: PONE-D-26-17082

Title: Disentangling Sensory Contributions to Postural Control Regulation Through Sample Entropy and Neural Modeling: a Preliminary Study Recommendation: Major Revision (Heavy Revision)Reviewer Assessment: Below average quality; significant flaws in methodology, presentation, and discussion; figures are poorly designed and non-informative.

Major Concerns

2.1 Extremely Small Sample Size & Missing Statistical Justification

Only 8 participants are included, which is acceptable for a preliminary study but no a priori sample size calculation or power analysis is provided (Lines 73–80). This violates PLOS ONE statistical reporting guidelines. N=8 is very small for a repeated-measures design with 3 visual states × 6 auditory/tactile conditions. High risk of Type II errors (false negatives), especially for interactions. Provide a post-hoc power analysis for the main effects observed.

We thank the reviewer for this careful consideration. We agree that our sample size is very small, and carefully revised the language in the paper to acknowledge this limitation and highlight that our work is preliminary. We also clarify that no a priori power calculation was performed due to the exploratory nature of the study and the limited availability of participants with the required experimental constraints. Furthermore, thanks to the reviewer suggestion, we have carefully revised the PLOS ONE statistical guideline, where the following is mentioned: "If a sample size calculation was performed, specify the inputs for power, effect size and alpha." We did not provide information about sample size calculation, therefore we did not report it as indicated by the journal's guidelines. Regarding post-hoc power analysis, we acknowledge the reviewer's suggestion. However, as discussed in the statistical literature (e.g., Zhang et al., 2019), post-hoc power is a direct transformation of observed p-values and does not provide additional information beyond effect sizes and confidence intervals. For this reason, we did not include such analyses. Instead, we report effect sizes to better characterize the magnitude of the observed effects and, as suggested, bootstrapped effect sizes Cis (see following responses).

Zhang, Y., Hedo, R., Rivera, A., Rull, R., Richardson, S., & Tu, X. M. (2019). Post hoc power analysis: is it an informative and meaningful analysis?. General psychiatry, 32(4), e100069.

Explicitly state in the abstract and discussion that findings are preliminary.

Thank you for the comment. Both the abstract and the discussion have been modified according to this comment to make it more explicit that we are introducing and discussing preliminary findings.

Consider using bootstrapped effect size CIs rather than relying solely on p-values.

Thank you for this suggestion. We now computed bootstrapped effect size CIs for each of our analyses.

The use of permutation ANOVA is appropriate for small samples, but: The manuscript does not specify whether permutations were within-subjects constrained (i.e., shuffling condition labels within each participant). If not, the test may be invalid for repeated measures. Clarify permutation scheme in Methods → Statistical analysis.

We thank the reviewer for the comment. Permutation tests were conducted using subject-constrained

permutations. Specifically, in the coin: independence_test mentioned in the Method section, participant ID was included as a blocking factor, ensuring that condition labels were permuted only within each participant and preserving the repeated-measures structure of the data. We added this information in the statistical analysis section.

The authors do not discuss how the tiny sample affects statistical reliability and generalizability.
Thank you for noticing this. We reviewed the discussion and, where appropriate, we made the necessary changes.

2.2 Severe Inconsistency Between Behavioral & Modeling Results (Unresolved)
Behavioral data show vibration INCREASES Sample Entropy (SamEn) (Lines 293–305), but the neural model shows vibration DECREASES SamEn (Lines 306–320).
The authors merely mention this discrepancy but provide no plausible neurophysiological explanation (Lines 351–370). This renders the model's validity questionable.
Thank you for pointing this out. We agree that this point needs a better clarification. First, It is important to note that the two entropy measures quantify fundamentally different dynamical systems and are not expected to vary in identical directions. We believe that the apparent discrepancy does not invalidate the model; rather, it represents one of its most compelling predictions. The model operates on the central representation of posture (i.e., the brainstem barycenter), rather than on the peripheral center of pressure (CoP). Achilles tendon's vibration exerts distinct effects at the peripheral and central levels:

1. ***Peripheral level:*** *tendon vibration introduces afferent sensory noise, thereby increasing the irregularity of the CoP signal and consequently increasing sample entropy.*
2. ***Central level:*** *in response to the degradation of proprioceptive input, the central nervous system (CNS) adopts a more rigid and stereotyped control strategy, resulting in more predictable brainstem dynamics and, consequently, a reduction in sample entropy.*

This framework is fully consistent with the complexity theory literature on motor control (Stergiou & Decker, 2011), which claims that movement variability reflects the structure of the control process rather than simple noise. This apparent paradox arises because the CNS shifts toward more conservative control strategies under conditions of sensory uncertainty. Reference Pakniyat & Namazi, 2021, already cited in the manuscript, further supports this interpretation.
We added a dedicated paragraph (lines 448-466) to discuss this point.

Stergiou, N., & Decker, L. M. (2011). *Human movement variability, nonlinear dynamics, and pathology: is there a connection?. Human movement science, 30(5), 869-888.*

Pakniyat, N., & Namazi, H. (2021). *Complexity-based analysis of the variations of brain and muscle reactions in walking and standing balance while receiving different perturbations. Frontiers in Human Neuroscience, 15, 749082.*

The model replicates some behavioral trends (blurred vision → lower entropy) but shows opposite effects for vibration (model: decreased entropy; behavior: increased entropy). The authors interpret this as a "divergence between peripheral and central entropy," but without direct neural recordings (EEG/fMRI), this remains speculation. Add a dedicated limitations paragraph acknowledging that model-behavior disagreement may indicate missing model components (e.g., spinal or muscle-level dynamics).

We thank the reviewer for this comment. We have modified the existing limitation section adding the suggested limitation related to the missing neural recordings. Limitations related to the missing descending pathways was already present in the section.

Show simulated CoP (not just brainstem barycenter) if possible, or explain why brainstem activity must correlate with CoP under all conditions.

Thank you. Explanations about brainstem activity correlation with CoP have been added in the Model simulation paragraph.

No behavioral effect of sound is found, but the model shows strong central effects. The conclusion that "dynamic auditory cues may have potential to impact CoP predictability" is speculative without: A control task where auditory cues are behaviorally relevant (e.g., spatial localization task). A more challenging balance task (e.g., Romberg, foam surface). Re-frame as hypothesis-generating, not evidence of a latent effect.

We thank the reviewer for the comment. The sentence has been reframed as requested.

"Predictability" is used to mean low Sample Entropy. Define explicitly in the introduction. "Rigid control strategy" (blurred vision) implies stiffness – this is a physiological claim not directly measured. Soften to "less flexible postural dynamics." Abstract says "first time to our knowledge" – this is fine but verify that no prior neural mass model of postural multisensory integration exists (e.g., work by Kuo, van der Kooij, or Chiba).

We thank the reviewer for the comment. These issues have been addressed throughout the manuscript where needed. We rephrased the last sentence of the abstract to cautiously specify that no prior neural mass model investigated multisensory integration with respect to postural control predictability. None of the models cited by the reviewer employs Sample Entropy as an output metric, nor do they model the integration of auditory and postural sensory information.

2.3 Insufficient & Non-Replicable Methodological Details

Visual blur manipulation: No quantification (e.g., visual acuity, light transmittance) (Lines 103–110). Other researchers cannot replicate the setup.

Thank you for noticing this. We added more information about how our blurring technique affected normal vision, (lines 138-145) so that others can replicate this setup more closely now.

Vibration parameters: 180 Hz vibration intensity, duration, and contact pressure are not reported (Lines xxx) in the method section. Sample Entropy parameters (m=4, r=0.25): No sensitivity test is presented to justify these values for CoP data (Lines 160–170).

We added information about vibration parameters in the manuscript. About Sample Entropy

parameters, we now report sensitivity analysis in the Supporting Information, Figure A2 and A3 in the Appendix A, and we refer to them in the manuscript.

Neural model parameters: Physiological justification for synaptic weights and noise scaling is missing (Lines 239–260).
We added a specific paragraph addressing this point (Lines 266-272 - Paragraph Model Simulation)

2.4 Poorly Designed & Uninformative Figures (Major Defect)
All figures are unpolished, low-quality, and missing standard statistical elements (Lines 288–320; Figures 1–4):
Figure 1: Simple schematic with no scale, no clear labeling of equipment, and low visual clarity.
Figure 3: Shows only individual data points without error bars (mean±SD/SEM); no statistical annotation; layout is messy and difficult to interpret.
Figure 4: Top panels: No error bars; significance asterisks are unclear.
Simulation panel: Y-axis scale is inconsistent; labels are vague; no units or statistical details.
General figure issues: No proper legends, no units, no definition of symbols; figures do not effectively support the results.
All figures must be completely redesigned and reconstructed to meet basic academic journal standards.
Thank you for these comments. All figures have now been redesigned to meet the academic journal standards as suggested.

2.5 Weak Neurophysiological Justification of the Model
The model excludes the vestibular system, which is essential for postural control (Lines 172–196). This is a major structural oversight.
Thank you for this comment. Although we strongly acknowledge that the vestibular system is integral to postural control, it was not manipulated across experimental conditions; all participants stood on a stable surface without galvanic vestibular stimulation or head perturbation. Therefore, vestibular input was modeled as a constant baseline drive to the cerebellar module, contributing to resting-state activity but not to condition-specific Sample Entropy differences. Its inclusion as a dynamic variable would have introduced a free parameter without empirical constraint from our data. This explanation is reported on lines 223-231 in the General model structure paragraph.

No model robustness test (parameter sensitivity analysis) is provided; results may be due to arbitrary parameter tuning.
We acknowledge that a formal parameter sensitivity analysis was not performed in the present study; this now constitutes a recognized limitation (page and lines), and future work will address it by adopting biologically validated neuro-musculoskeletal models of the spinal-peripheral effector stage (such as those developed by Elias and colleagues) that would also provide the physiological constraints needed to anchor parameter ranges more tightly. Nevertheless, as stated in paragraph General model structure, lines 209-210, parameters were tuned based on physiologically available information (see references [2,14,35,36] in the manuscript.

Elias LA, Watanabe RN, Kohn AF. Spinal Mechanisms May Provide a Combination of Intermittent and Continuous Control of Human Posture: Predictions from a Biologically Based Neuromusculoskeletal Model. PLoS Comput

Biol 2014;10.

[2]Kandel ER, Schwartz JH, Jessell TM, Siegelbaum S, Hudspeth AJ, Mack S. Principles of neural science. 5th ed. New York: McGraw-Hill: 2000.

[14] Takakusaki K. Functional Neuroanatomy for Posture and Gait Control. J Mov Disord 2017;10:1–17. https://doi.org/10.14802/jmd.16062.

[35] Ramnani N. The primate cortico-cerebellar system: anatomy and function 2006;7:511–22. https://doi.org/10.1038/nrn1953.

[36] Horak FB, Macpherson JM. Postural orientation and equilibrium, 1996.

The model does not include descending motor pathways, so the link between central neural activity and CoP behavior is unsupported (Lines 401–410).
This has been clarified in the Discussion, specifically at the end of Proprioception modulation paragraph.

2.6 Superficial Discussion & Limited Novelty
The study is a simple combination of existing techniques (sample entropy + neural mass model) with no novel conceptual contribution.
Thank you for pointing this out. The novelty of the present work does not lie in the simple combination of sample entropy analysis with a neural mass model. Rather, it resides in the first application of a neurocomputational model to predict the sample entropy of central neural activity within a postural paradigm involving multisensory integration. None of the models previously mentioned by the reviewer (Kuo, van der Kooij, or Chiba) employs Sample Entropy as an output metric, nor do they model the integration of auditory and postural sensory information. Accordingly, the novelty of the proposed approach stems from the development of a computational framework capable of linking central neural dynamics, quantified through entropy-based measures, with multisensory mechanisms underlying postural control. We added a short sentence to clarify this novel aspect of the present work [Discussion paragraph].

The “auditory central modulation” finding is weakly supported and not placed in the context of recent multisensory integration literature.
Thank you. More literature about auditory central modulation has been added to the discussion.

Clinical implications are entirely missing; the translational value is not discussed.
Thank you. We added a paragraph about clinical implications after the main Discussion section.

3. Minor Concerns
Abbreviation errors: CoP, SamEn, SOT, AP, ML are not consistently defined at first use.
Writing quality: Poor grammar, awkward sentence structure, and disorganized logic; professional English editing is required.
We revised the whole manuscript accordingly.

Data transparency: Data availability states “will be available upon acceptance” but provides no repository link or data dictionary (Lines 426–430).
We added the link of the repository to our statement, which however will remain not accessible pending Institutional approval.

Limitations: The authors ignore key limitations (e.g., no dynamic postural tasks, no clinical population validation).
We improved the limitation section according to reviewer concerns.

4. Recommendation
Major Revision
All identified flaws require substantial revision. If thoroughly revised (including full reconstruction of figures, resolution of result discrepancies, and improved mechanistic interpretation), the manuscript may be suitable for publication in PLOS ONE.
