# Supplementary material for: Disentangling sensory contributions to postural control regulation through sample entropy and neural modeling: A preliminary study
Source: PLoS One. 2026 Aug 18;21(8):e0355402. doi: 10.1371/journal.pone.0355402 (PMC13485045; doi:10.1371/journal.pone.0355402)
Supplement: Supplementary file 2 [file pone.0355402.s001.docx]

Supporting information

**Table S1. Alpha values before and after adjustment for long-range correlation**. Calculation of mean α values of Anterior-posterior (AP) and Medio-lateral (ML) time series before and after adjusting data for long-range correlations. Values in brackets represent the standard deviation of α for each group of data.

| **Spatial Domain** | **α before adjustment for long-range correlation** | **α after adjustment for long-range correlation** |
| --- | --- | --- |
| Anterior-Posterior (AP) | 1.15 (±0.17) | 0.48 (±0.14) |
| Medio-Lateral (ML) | 1.05 (±0.18) | 0.37 (±0.12) |

**Figure S1.** **Detrended Fluctuation Analysis before and after correction.** Example of a log-log plot of a random time-series within our dataset before and after correcting for long-range correlation. The x-axis of the figures represents the scale (log), i.e., the size of the window or segment of the data. The y-axis represents the fluctuation (log), which corresponds to the root-mean-square deviation of the detrended data in each window. The points (red circles) represent the calculated fluctuation values at each scale. A) Example of a time-series: clear linear relationship in the log-log scale indicates a power-law relationship, with α= 1.105 describing the correlation structure. B) Time-series after adjusting for long-range correlation: α= 0.500: This scaling exponent suggests the data behaves like white noise (uncorrelated random data), indicating the data is closer to uncorrelated noise.

**Figure S2. Selection of embedding dimensions (m) for AP and ML planes of CoP.** Varying m {1,2...5} in function of different tolerance radius (r) {0.05,0.10,0.15,…,1}show consistent convergence behavior across both AP and ML planes of the CoP signal. As *m* increases, the Sample Entropy estimates stabilize, with minimal differences observed beyond *m=3*. This indicates that *m ≥ 3* is an optimal choice, balancing computational efficiency and reliable convergence for both planes.

**Figure S3. Selection of tolerance radius (r):** Heatmaps of the maximum relative error Q(m,r) of Sample Entropy and conditional probability estimates in the AP and ML planes, following the approach described by (Ramdani et al. 2009). The tolerance radius r is selected such that the targeted maximum relative error remains below 0.05, ensuring reliable estimation. According to this criterion, maintaining Q(m,r)<0.05 guarantees a 95% confidence interval with a width not exceeding 10% of the entropy estimate. The results indicate that small r values yield high estimation errors, especially for low embedding dimensions (m = 1–2), while stable and acceptable error levels are achieved for moderate r values (≈0.2–0.4) and m ≥ 3, supporting robust parameter selection.

**Eq. S1: Mathematical definition of neurons nonlinear activation function.**

$$x_{\sigma}^{{}^{j}}\left( k+1 \right) = x_{\sigma}^{{}^{j}}\left( k \right)+\Delta t\left( G\cdot\frac{1}{\tau}\cdot\left( - x_{\sigma}^{{}^{j}}\left( k \right)+\frac{1}{1+e^{-\left( u_{j}\left( k \right)-\varphi\right)\cdot pend}} \right) \right)$$

- $x_{\sigma}^{j}$ represents the current state of neuron activation.
- ∆t=0.1 is the time step.
- G=1 is the gain factor.
- τ =3ms is the time constant.
- $u_{j}$ is the overall input at time step k.
- φ=20 is the threshold parameter.
- pend = 0.2 is a scaling factor affecting the steepness of the sigmoid function.

**Eq. S2: Mathematical expression of the feedforward and feedback synapses designed based on a Gaussian distribution.**

$$W_{ij} = W_{ij0} \cdot e^{-\left( \frac{d_{ij}}{2\sigma\sigma_{ij}} \right)}$$

- $W_{ij0}$ is the maximum synaptic weight.
- $d_{ij}$ represents the distance between neuron *i* and neuron *j*, ensuring that neurons farther from the central one receive lower synaptic weights.
- $\sigma_{ij}$ is the standard deviation of the Gaussian function, determining the spread of connectivity.

**Eq. S3: Mathematical formulation of the lateral and inter-area synapses following a Gaussian distribution.** These are organized in a circular structure accordingly to the Mexican-Hat configuration, ensuring that local interactions are stronger than distant ones.

$$L_{lat}\left( i,j \right) = L_{0}^{ex} \cdot e^{-\left( \frac{d_{l}\left( i,j \right)}{2\sigma\sigma_{ex}} \right)} - L_{0}^{in} \cdot e^{-\left( \frac{d_{l}\left( i,j \right)}{2\sigma\sigma_{in}} \right)}$$

- $L_{lat}\left( i,j \right)$ is the weight of the lateral synapse between neuron *i* and neuron *j*.
- $L_{0}^{ex}$ and $L_{0}^{in}$ are the maximum excitatory and inhibitory synaptic weights,	respectively.
- $\sigma_{ex}$	and $\sigma_{in}$ are the standard deviations of the Gaussian distribution governing	excitation and inhibition, respectively.
- $d_{l}\left( i,j \right)$ represents the distance between neuron *i* and neuron *j*, ensuring that	synaptic strength decreases with increasing distance, with closer neurons having stronger connections.

In this case as well, the lateral synapses within each brain region are represented by an *(NxN)* matrix. Self-connections are set to zero, ensuring that a neuron does not influence itself. The net effect of lateral synapses is determined by subtracting the excitatory component from the inhibitory one. Notably, the standard deviation of excitatory connections is narrower than that of inhibitory connections, and excitatory synapses are generally weaker. As a result, excitatory connections are more localized, mainly affecting nearby neurons; inhibitory connections have a wider spread, influencing more distant neurons. This arrangement establishes a competitive dynamic, where neighboring neurons reinforce each other through excitation, while more distant neurons exert inhibition, promoting the efficiency of the network.

**Eq. S4: Mathematical computation of the Sample Entropy of the barycenter of the brainstem activity.**

$$b = \frac{\Sigma_{i}\left( x_{i} \cdot n_{i} \right)}{\Sigma_{i} x_{i}}$$

- $x_{i}$	represents the neural activity.
- $n_{i}$	denotes the neuron’s position.
- $b$	is the barycenter

**Table S2. Feedforward (from column 2 to 5) and feedback synapses (last column) connecting the neural network areas.** SC = superior colliculus.

|  | **Cerebellum - Motor cortex** |  | **Parietal Cortex - Motor cortex** | **Parietal Cortex - SC** | **Motor Cortex & SC - Brainstem** | **Feedback Synapses** |
| --- | --- | --- | --- | --- | --- | --- |
| $W_{ij0}$ | 2,5  1  0.02 | | 2,5  1  0.02 | 5  1  0.01 | 5  1  0.01 | 3  1  0.01 |
| $\sigma_{ij}$ |  |  |  |  |  |  |
| $\sigma_{noise}$ |  |  |  |  |  |  |

**Table S3.** **Lateral synapses (left) and inter-area synapses (right).** SC = superior colliculus.

|  | **Lateral synapses** | **Inter-area synapses** |
| --- | --- | --- |
| $L_{0}^{ex}$ | 0.09  0.06  3  90  0.12 | 0.09  0.1  1  120  0.8 |
| $L_{0}^{in}$ |  |  |
| $\sigma_{ex}$ |  |  |
| $\sigma_{in}$ |  |  |
| $\sigma_{noise}$ |  |  |

**Table S4.** **Input intensities in the experimental and in the SOT paradigm.** SOT = Sensory Organization Test

|  | **Experimental**  **Paradigm** | **SOT**  **Paradigm** |
| --- | --- | --- |
| **Proprioceptive input intensity** | 15 | 30 |
| **Vestibular input intensity** | 10 | 15 |
| **Visual input intensity** | 7 | 10 |
| **Auditory input intensity** | 9 | 0 |

**D**

**C**

**B**

**A**

**Figure S4.** **DHARMa residual diagnostics for our model on Sample Entropy data in AP, ML and simulated data.** The QQ plots for AP, ML and SOT model (**panels A, B, D**, respectively) indicate no deviation from normality with Kolmogorov-Smirnov test; tests for dispersion and outlier are not significant. The QQ plot for the simulated model (**panel C**) indicates mild tail deviations and a few extreme residuals; uniformity and outlier tests are significant, while dispersion is not. No structure is visible in residuals vs. fitted values. Globally, the observed deviations are not gross and are not expected to compromise repeated‑measures ANOVA validity.

Table S5. Statistical results from permutation‑based paired comparisons (n=10000 permutations) performed on the simulated dataset. Three families of contrasts were tested: (A) Vibration ON vs OFF within each combination of visual state and sound level. (B) Pairwise comparisons among visual states (eyes open, blurred vision, eyes closed) within each combination of sound level and vibration level. (C) Pairwise comparisons among sound levels (none, static, moving) within each combination of visual state and vibration level. All p‑values are Bonferroni‑corrected. As mentioned in the manuscript, 95% confidence intervals for the paired mean differences between conditions were estimated using bias-corrected and accelerated (BCa) bootstrap resampling at the participant level.

A)

| **EYES OPEN -Comparison** | **p** | **Hedges’ g** | **Mean difference** | **CI Lower** | | **CI Upper** |
| --- | --- | --- | --- | --- | --- | --- |
| **Sound = none** |  |  |  | |  |  |
| Vibration OFF - ON | .0249 | 3.36 | -0.00592 | | -0.00732 | -0.00498 |
| **Sound = Sound Moving** |  |  |  | |  |  |
| Vibration OFF - ON | .0255 | 1.82 | -0.00235 | | -0.00318 | -0.00154 |
| **Sound = Sound Static** |  |  |  | |  |  |
| Vibration OFF - ON | .0228 | 3.16 | -0.00616 | | -0.00764 | -0.00504 |

BLURRED VISION-Comparison

| **Sound = none** |  |  |  |  |  |
| --- | --- | --- | --- | --- | --- |
| Vibration OFF - ON | .0258 | 4.74 | -0.00769 | -0.00916 | -0.00691 |
| **Sound = Sound Moving** |  |  |  |  |  |
| Vibration OFF - ON | .0255 | 2.10 | -0.00450 | -0.00661 | -0.00350 |
| **Sound = Sound Static** |  |  |  |  |  |
| Vibration OFF - ON | .0231 | 4.29 | -0.00953 | -0.0117 | -0.00847 |
| **EYES CLOSED-Comparison** |  |  |  |  |  |
| **Sound = none** |  |  |  |  |  |
| Vibration OFF - ON | .0246 | 3.35 | -0.00546 | -0.00673 | -0.00458 |
| **Sound = Sound Moving** |  |  |  |  |  |
| Vibration OFF - ON | .024 | 1.70 | -0.00212 | -0.00297 | -0.00137 |
| **Sound = Sound Static** |  |  |  |  |  |
| Vibration OFF - ON | .0255 | 2.90 | -0.00552 | -0.00684 | -0.00436 |

**B)**

| **NO SOUND -Comparison** | **p** | **Hedges’ g** | **Mean difference** | **CI Lower** | **CI Upper** |
| --- | --- | --- | --- | --- | --- |
| **Vibration = OFF** |  |  |  |  |  |
| Eyes Open – Blurred Vision | .0243 | -5.7 | -0.00415 | -0.00463 | -0.00370 |
| Blurred Vision – Eyes Closed | .0255 | 4.37 | 0.00447 | 0.00376 | 0.00506 |
| Eyes Open – Eyes Closed | .169 | 0.88 | 0.000321 | -0.0000151 | 0.000492 |
| **Vibration = ON** |  |  |  |  |  |
| Eyes Open – Blurred Vision | .0255 | -8.44 | -0.00592 | -0.00637 | -0.00547 |
| Blurred Vision – Eyes Closed | .0234 | 6.47 | 0.00670 | 0.00599 | 0.00734 |
| Eyes Open – Eyes Closed | .0219 | 1.8 | 0.000778 | 0.000487 | 0.00105 |

| **SOUND STATIC -Comparison** | **p** | **Hedges’ g** | **Mean difference** | **CI Lower** | **CI Upper** |
| --- | --- | --- | --- | --- | --- |
| **Vibration = OFF** |  |  |  |  |  |
| Eyes Open – Blurred Vision | .021 | -7.04 | -0.00436 | -0.00477 | -0.00397 |
| Blurred Vision – Eyes Closed | .018 | 7.03 | 0.00490 | 0.00446 | 0.00536 |
| Eyes Open – Eyes Closed | .0249 | 2.75 | 0.000534 | 0.000409 | 0.000661 |
| **Vibration = ON** |  |  |  |  |  |
| Eyes Open – Blurred Vision | .0249 | -7.85 | -0.00773 | -0.00844 | -0.00717 |
| Blurred Vision – Eyes Closed | .0246 | 8.33 | 0.00891 | 0.00827 | 0.00964 |
| Eyes Open – Eyes Closed | .0273 | 5.78 | 0.00118 | 0.00105 | 0.00132 |

| **SOUND MOVING -Comparison** | **p** | **Hedges’ g** | **Mean difference** | **CI Lower** | **CI Upper** |
| --- | --- | --- | --- | --- | --- |
| **Vibration = OFF** |  |  |  |  |  |
| Eyes Open – Blurred Vision | .024 | -5.93 | -0.00319 | -0.00353 | -0.00283 |
| Blurred Vision – Eyes Closed | .0267 | 5.62 | 0.00357 | 0.00319 | 0.00401 |
| Eyes Open – Eyes Closed | .0186 | 2.21 | 0.000379 | 0.000271 | 0.000494 |
| **Vibration = ON** |  |  |  |  |  |
| Eyes Open – Blurred Vision | .0261 | -4.33 | -0.00534 | -0.00641 | -0.00476 |
| Blurred Vision – Eyes Closed | .0246 | 4.81 | 0.00594 | 0.00530 | 0.00699 |
| Eyes Open – Eyes Closed | .0243 | 3.88 | 0.000606 | 0.000514 | 0.000714 |

**C)**

| **EYES OPEN -Comparison** | **p** | | **Hedges’ g** | **Mean difference** | **CI Lower** | **CI Upper** |
| --- | --- | --- | --- | --- | --- | --- |
| **Vibration = OFF** |  | |  |  |  |  |
| None – Sound Static | 0.167 | | 0.681 | 0.00108 | 0.000391 | 0.00260 |
| None – Sound Moving | .0285 | | 1.14 | 0.00127 | 0.000701 | 0.00272 |
| Sound Static – Sound Moving | .0264 | | 1.15 | 0.000196 | 0.000059 | 0.000489 |
| **Vibration = ON** |  | |  |  |  |  |
| None – Sound Static | .239 | | 0.56 | 0.00102 | 0.000451 | 0.00253 |
| None – Sound Moving | .0249 | | 2.96 | 0.00462 | 0.00381 | 0.00647 |
| Sound Static – Sound Moving | .0264 | | 7.41 | 0.00360 | 0.00322 | 0.00396 |
| **BLURRED VISION -Comparison** | | **p** | **Hedges’ g** |  | **CI Lower** | **CI Upper** |
| **Vibration = OFF** |  | |  |  |  |  |
| None – Sound Static | 0.022 | | 1.10 | 0.000650 | 0.000361 | 0.00119 |
| None – Sound Moving | .025 | | 3.39 | 0.00218 | 0.00190 | 0.00283 |
| Sound Static – Sound Moving | .021 | | 5.49 | 0.00153 | 0.00138 | 0.00175 |
| **Vibration = ON** |  | |  |  |  |  |
| None – Sound Static | .0267 | | -3.68 | -0.00119 | -0.00138 | -0.000966 |
| None – Sound Moving | .0231 | | 14.5 | 0.00537 | 0.00511 | 0.00559 |
| Sound Static – Sound Moving | .0303 | | 31.3 | 0.00656 | 0.00642 | 0.00670 |

| **EYES CLOSED -Comparison** | **p** | **Hedges’ g** | **Mean difference** | **CI Lower** | **CI Upper** |
| --- | --- | --- | --- | --- | --- |
| **Vibration = OFF** |  |  |  |  |  |
| None – Sound Static | .13 | 0.716 | 0.00108 | 0.000404 | 0.00262 |
| None – Sound Moving | .0204 | 0.957 | 0.00127 | 0.000700 | 0.00268 |
| Sound Static – Sound Moving | .206 | 0.669 | 0.000196 | 0.0000567 | 0.000474 |
| **Vibration = ON** |  |  |  |  |  |
| None – Sound Static | .0483 | 0.738 | 0.00102 | 0.000424 | 0.00244 |
| None – Sound Moving | .0225 | 2.56 | 0.00462 | 0.00382 | 0.00644 |
| Sound Static – Sound Moving | .0231 | 6.33 | 0.00360 | 0.00322 | 0.00396 |
