# Supplementary material for: Disentangling sensory contributions to postural control regulation through sample entropy and neural modeling: A preliminary study
Source: PLoS One. 2026 Aug 18;21(8):e0355402. doi: 10.1371/journal.pone.0355402 (PMC13485045; doi:10.1371/journal.pone.0355402)
Supplement: Supplementary file 3 [file pone.0355402.s002.docx]

Manuscript ID: PONE-D-26-17082

Title: Disentangling Sensory Contributions to Postural Control Regulation Through Sample Entropy and Neural Modeling: a Preliminary StudyRecommendation: Major Revision (Heavy Revision)Reviewer Assessment: Below average quality; significant flaws in methodology, presentation, and discussion; figures are poorly designed and non-informative.

1. Overall Evaluation

This is a preliminary exploratory study that combines sample entropy analysis and neurocomputational modeling to investigate multisensory integration in postural control. The topic is marginally relevant to PLOS ONE’s scope, and the core experimental logic is generally valid.

However, the overall manuscript quality is below average:

Novelty is limited (simple combination of existing methods with no theoretical or methodological breakthrough);

Key behavioral and modeling results are contradictory and poorly interpreted;

Methodological details are insufficient for replication;

Figures are poorly designed, unpolished, and lack essential statistical annotations;

The discussion is superficial and fails to explain critical mechanistic inconsistencies.

All flaws are reviseable but require substantial revision. Rejection is not recommended only because the study addresses a reasonable scientific question with acceptable basic methodology.

2. Major Concerns

2.1 Extremely Small Sample Size & Missing Statistical Justification

Only 8 participants are included, which is acceptable for a preliminary study but no a priori sample size calculation or power analysis is provided (Lines 73–80). This violates PLOS ONE statistical reporting guidelines.

The authors do not discuss how the tiny sample affects statistical reliability and generalizability.

2.2 Severe Inconsistency Between Behavioral & Modeling Results (Unresolved)

Behavioral data show vibration INCREASES Sample Entropy (SamEn) (Lines 293–305), but the neural model shows vibration DECREASES SamEn (Lines 306–320).

The authors merely mention this discrepancy but provide no plausible neurophysiological explanation (Lines 351–370). This renders the model’s validity questionable.

2.3 Insufficient & Non-Replicable Methodological Details

Visual blur manipulation: No quantification (e.g., visual acuity, light transmittance) (Lines 103–110). Other researchers cannot replicate the setup.

Vibration parameters: 180 Hz vibration intensity, duration, and contact pressure are not reported (Lines 93–100).

SamEn parameters (m=4, r=0.25): No sensitivity test is presented to justify these values for CoP data (Lines 160–170).

Neural model parameters: Physiological justification for synaptic weights and noise scaling is missing (Lines 239–260).

2.4 Poorly Designed & Uninformative Figures (Major Defect)

All figures are unpolished, low-quality, and missing standard statistical elements (Lines 288–320; Figures 1–4):

Figure 1: Simple schematic with no scale, no clear labeling of equipment, and low visual clarity.

Figure 3: Shows only individual data points without error bars (mean±SD/SEM); no statistical annotation; layout is messy and difficult to interpret.

Figure 4:

Top panels: No error bars; significance asterisks are unclear.

Simulation panel: Y-axis scale is inconsistent; labels are vague; no units or statistical details.

General figure issues: No proper legends, no units, no definition of symbols; figures do not effectively support the results.

All figures must be completely redesigned and reconstructed to meet basic academic journal standards.

2.5 Weak Neurophysiological Justification of the Model

The model excludes the vestibular system, which is essential for postural control (Lines 172–196). This is a major structural oversight.

No model robustness test (parameter sensitivity analysis) is provided; results may be due to arbitrary parameter tuning.

The model does not include descending motor pathways, so the link between central neural activity and CoP behavior is unsupported (Lines 401–410).

2.6 Superficial Discussion & Limited Novelty

The study is a simple combination of existing techniques (sample entropy + neural mass model) with no novel conceptual contribution.

The “auditory central modulation” finding is weakly supported and not placed in the context of recent multisensory integration literature.

Clinical implications are entirely missing; the translational value is not discussed.

3. Minor Concerns

Abbreviation errors: CoP, SamEn, SOT, AP, ML are not consistently defined at first use.

Writing quality: Poor grammar, awkward sentence structure, and disorganized logic; professional English editing is required.

Data transparency: Data availability states “will be available upon acceptance” but provides no repository link or data dictionary (Lines 426–430).

Limitations: The authors ignore key limitations (e.g., no dynamic postural tasks, no clinical population validation).

4. Recommendation

Major Revision

All identified flaws require substantial revision. If thoroughly revised (including full reconstruction of figures, resolution of result discrepancies, and improved mechanistic interpretation), the manuscript may be suitable for publication in PLOS ONE.
