# Supplementary material for: Disentangling sensory contributions to postural control regulation through sample entropy and neural modeling: A preliminary study
Source: PLoS One. 2026 Aug 18;21(8):e0355402. doi: 10.1371/journal.pone.0355402 (PMC13485045; doi:10.1371/journal.pone.0355402)
Supplement: Peer review history

# Peer review: Decision Letter 0

**DOI:** 10.1371/journal.pone.0355402.r001

**Authors:** Yih-Kuen Jan

26 Apr 2026

Dear Dr. Montagnani,

Thank you for submitting your manuscript to PLOS ONE. After careful consideration, we feel that it has merit but does not fully meet PLOS ONE’s publication criteria as it currently stands. Therefore, we invite you to submit a revised version of the manuscript that addresses the points raised during the review process.

Please submit your revised manuscript by Jun 10 2026 11:59PM. If you will need more time than this to complete your revisions, please reply to this message or contact the journal office at plosone@plos.org. When you're ready to submit your revision, log on to https://www.editorialmanager.com/pone/ and select the 'Submissions Needing Revision' folder to locate your manuscript file.

- A letter that responds to each point raised by the academic editor and reviewer(s). You should upload this letter as a separate file labeled 'Response to Reviewers'.
- A marked-up copy of your manuscript that highlights changes made to the original version. You should upload this as a separate file labeled 'Revised Manuscript with Track Changes'.
- An unmarked version of your revised paper without tracked changes. You should upload this as a separate file labeled 'Manuscript'.

If you would like to make changes to your financial disclosure, please include your updated statement in your cover letter. Guidelines for resubmitting your figure files are available below the reviewer comments at the end of this letter.

If applicable, we recommend that you deposit your laboratory protocols in protocols.io to enhance the reproducibility of your results. Protocols.io assigns your protocol its own identifier (DOI) so that it can be cited independently in the future. For instructions see: https://journals.plos.org/plosone/s/submission-guidelines#loc-laboratory-protocols. Additionally, PLOS ONE offers an option for publishing peer-reviewed Lab Protocol articles, which describe protocols hosted on protocols.io. Read more information on sharing protocols at https://plos.org/protocols?utm_medium=editorial-email&utm_source=authorletters&utm_campaign=protocols.

As the corresponding author, your ORCID iD is verified in the submission system and will appear in the published article. PLOS supports the use of ORCID, and we encourage all coauthors to register for an ORCID iD and use it as well. Please encourage your coauthors to verify their ORCID iD within the submission system before final acceptance, as unverified ORCID iDs will not appear in the published article. *Only*  the individual author can complete the verification step; PLOS staff *cannot*  verify ORCID iDs on behalf of authors.

We look forward to receiving your revised manuscript.

Kind regards,

Yih-Kuen Jan, PhD

Academic Editor

PLOS One

Journal Requirements:

When submitting your revision, we need you to address these additional requirements.

1. Please ensure that your manuscript meets PLOS ONE's style requirements, including those for file naming. The PLOS ONE style templates can be found at

https://journals.plos.org/plosone/s/file?id=wjVg/PLOSOne_formatting_sample_main_body.pdf and

https://journals.plos.org/plosone/s/file?id=ba62/PLOSOne_formatting_sample_title_authors_affiliations.pdf

2. Thank you for stating the following in the Acknowledgments Section of your manuscript:

“The research was partially supported by the MYSpace project (principal investigator Monica Gori), which has received funding from the European Research Council under the European Union’s Horizon 2020 research and innovation program (Grant 948349) and by the Brain and Machines Flagship Programme of the Italian Institute of Technology.”

We note that you have provided funding information that is not currently declared in your Funding Statement. However, funding information should not appear in the Acknowledgments section or other areas of your manuscript. We will only publish funding information present in the Funding Statement section of the online submission form.

Please remove any funding-related text from the manuscript and let us know how you would like to update your Funding Statement. Currently, your Funding Statement reads as follows:

“The author(s) received no specific funding for this work.”

Please include your amended statements within your cover letter; we will change the online submission form on your behalf.

3. Thank you for stating the following financial disclosure:

“The research was partially supported by the MYSpace project (principal investigator Monica Gori), which has received funding from the European Research Council under the European Union’s Horizon 2020 research and innovation program (Grant 948349) and by the Brain and Machines Flagship Programme of the Italian Institute of Technology.”

At this time, please address the following queries:

a) Please clarify the sources of funding (financial or material support) for your study. List the grants or organizations that supported your study, including funding received from your institution.

b) State what role the funders took in the study. If the funders had no role in your study, please state: “The funders had no role in study design, data collection and analysis, decision to publish, or preparation of the manuscript.”

c) If any authors received a salary from any of your funders, please state which authors and which funders.

d) If you did not receive any funding for this study, please state: “The authors received no specific funding for this work.”

Please include your amended statements within your cover letter; we will change the online submission form on your behalf.

4. Thank you for stating in your Funding Statement:

“The research was partially supported by the MYSpace project (principal investigator Monica Gori), which has received funding from the European Research Council under the European Union’s Horizon 2020 research and innovation program (Grant 948349) and by the Brain and Machines Flagship Programme of the Italian Institute of Technology.”

Please provide an amended statement that declares *all* the funding or sources of support (whether external or internal to your organization) received during this study, as detailed online in our guide for authors at http://journals.plos.org/plosone/s/submit-now.  Please also include the statement “There was no additional external funding received for this study.” in your updated Funding Statement.

Please include your amended Funding Statement within your cover letter. We will change the online submission form on your behalf.

5. When completing the data availability statement of the submission form, you indicated that you will make your data available on acceptance. We strongly recommend all authors decide on a data sharing plan before acceptance, as the process can be lengthy and hold up publication timelines. Please note that, though access restrictions are acceptable now, your entire data will need to be made freely accessible if your manuscript is accepted for publication. This policy applies to all data except where public deposition would breach compliance with the protocol approved by your research ethics board. If you are unable to adhere to our open data policy, please kindly revise your statement to explain your reasoning and we will seek the editor's input on an exemption. Please be assured that, once you have provided your new statement, the assessment of your exemption will not hold up the peer review process.

6. Please include captions for your Supporting Information files at the end of your manuscript, and update any in-text citations to match accordingly. Please see our Supporting Information guidelines for more information: http://journals.plos.org/plosone/s/supporting-information.

7. If the reviewer comments include a recommendation to cite specific previously published works, please review and evaluate these publications to determine whether they are relevant and should be cited. There is no requirement to cite these works unless the editor has indicated otherwise.

[Note: HTML markup is below. Please do not edit.]

Reviewers' comments:

Reviewer's Responses to Questions

**Comments to the Author**

1. Is the manuscript technically sound, and do the data support the conclusions?

Reviewer #1: Yes

Reviewer #2: Yes

**********

2. Has the statistical analysis been performed appropriately and rigorously?

Reviewer #1: No

Reviewer #2: Yes

**********

3. Have the authors made all data underlying the findings in their manuscript fully available?

The PLOS Data policy

Reviewer #1: Yes

Reviewer #2: Yes

**********

4. Is the manuscript presented in an intelligible fashion and written in standard English?

Reviewer #1: Yes

Reviewer #2: Yes

**********

Reviewer #1: This study combines a human balance experiment with a neural mass model to examine how visual, proprioceptive, and auditory manipulations affect postural control predictability (Sample Entropy). The behavioral finding that Achilles’ tendon vibration increases CoP entropy is robust. The modeling work is ambitious and valuable, but several methodological and interpretational issues need addressing.

N=8 is very small for a repeated-measures design with 3 visual states × 6 auditory/tactile conditions. High risk of Type II errors (false negatives), especially for interactions. Provide a post-hoc power analysis for the main effects observed. Explicitly state in the abstract and discussion that findings are preliminary. Consider using bootstrapped effect size CIs rather than relying solely on p-values.

The use of permutation ANOVA is appropriate for small samples, but: The manuscript does not specify whether permutations were within-subjects constrained (i.e., shuffling condition labels within each participant). If not, the test may be invalid for repeated measures. Clarify permutation scheme in Methods → Statistical analysis.

The model replicates some behavioral trends (blurred vision → lower entropy) but shows opposite effects for vibration (model: decreased entropy; behavior: increased entropy). The authors interpret this as a “divergence between peripheral and central entropy,” but without direct neural recordings (EEG/fMRI), this remains speculation. Add a dedicated limitations paragraph acknowledging that model-behavior disagreement may indicate missing model components (e.g., spinal or muscle-level dynamics). Show simulated CoP (not just brainstem barycenter) if possible, or explain why brainstem activity must correlate with CoP under all conditions.

No behavioral effect of sound is found, but the model shows strong central effects. The conclusion that “dynamic auditory cues may have potential to impact CoP predictability” is speculative without: A control task where auditory cues are behaviorally relevant (e.g., spatial localization task). A more challenging balance task (e.g., Romberg, foam surface). Re-frame as hypothesis-generating, not evidence of a latent effect.

“Predictability” is used to mean low Sample Entropy. Define explicitly in the introduction. “Rigid control strategy” (blurred vision) implies stiffness – this is a physiological claim not directly measured. Soften to “less flexible postural dynamics.” Abstract says “first time to our knowledge” – this is fine but verify that no prior neural mass model of postural multisensory integration exists (e.g., work by Kuo, van der Kooij, or Chiba).

Reviewer #2: This manuscript reports a methodologically innovative and valuable preliminary study that well meets PLOS ONE’s publication criteria for scientific validity, standard methodology, and incremental academic contribution. The study addresses an important research gap by investigating multisensory integration in postural control using a novel combination of multi-modal sensory perturbations, sample entropy analysis, and neurocomputational modeling. Its key finding—revealing the hidden central modulation of auditory inputs on postural regulation—represents a meaningful addition to the literature.

The methodological design is generally rigorous: permutation ANOVA is appropriately adopted for the small sample, and the neural model is reliably validated using the Sensory Organization Test (SOT), showing good logical consistency. This work provides a new integrated approach for evaluating postural control and carries clear translational potential for future research in aging and balance-impaired populations.

All concerns raised are minor-to-moderate and fully correctable with targeted revisions; no fatal design flaws or critical scientific issues are present. Major Revision is therefore recommended to further improve methodological transparency, result interpretation, figure quality, and presentation clarity.

**********

what does this mean?). If published, this will include your full peer review and any attached files.

If you choose “no”, your identity will remain anonymous but your review may still be made public.

**Do you want your identity to be public for this peer review?**  For information about this choice, including consent withdrawal, please see our Privacy Policy

Reviewer #1: **Yes:**  Yes

Reviewer #2: **Yes:**  Lingyue Meng

**********

[NOTE: If reviewer comments were submitted as an attachment file, they will be attached to this email and accessible via the submission site. Please log into your account, locate the manuscript record, and check for the action link "View Attachments". If this link does not appear, there are no attachment files.]

To ensure your figures meet our technical requirements, please review our figure guidelines: https://journals.plos.org/plosone/s/figures

You may also use PLOS’s free figure tool, NAAS, to help you prepare publication quality figures: https://journals.plos.org/plosone/s/figures#loc-tools-for-figure-preparation.

NAAS will assess whether your figures meet our technical requirements by comparing each figure against our figure specifications.

Attachment Submitted filename: C.docx

# Author comment: Author response to Decision Letter 1

**DOI:** 10.1371/journal.pone.0355402.r002

6 Jul 2026

Major Concerns

2.1 Extremely Small Sample Size & Missing Statistical Justification

Only 8 participants are included, which is acceptable for a preliminary study but no a priori sample size calculation or power analysis is provided (Lines 73–80). This violates PLOS ONE statistical reporting guidelines. N=8 is very small for a repeated-measures design with 3 visual states × 6 auditory/tactile conditions. High risk of Type II errors (false negatives), especially for interactions. Provide a post-hoc power analysis for the main effects observed.

We thank the reviewer for this careful consideration. We agree that our sample size is very small, and carefully revised the language in the paper to acknowledge this limitation and highlight that our work is preliminary. We also clarify that no a priori power calculation was performed due to the exploratory nature of the study and the limited availability of participants with the required experimental constraints. Furthermore, thanks to the reviewer suggestion, we have carefully revised the PLOS ONE statistical guideline, where the following is mentioned: "If a sample size calculation was performed, specify the inputs for power, effect size and alpha." We did not provide information about sample size calculation, therefore we did not report it as indicated by the journal's guidelines.

Regarding post-hoc power analysis, we acknowledge the reviewer’s suggestion. However, as discussed in the statistical literature (e.g., Zhang et al., 2019), post-hoc power is a direct transformation of observed p-values and does not provide additional information beyond effect sizes and confidence intervals. For this reason, we did not include such analyses. Instead, we report effect sizes to better characterize the magnitude of the observed effects and, as suggested, bootstrapped effect sizes Cis (see following responses).

Zhang, Y., Hedo, R., Rivera, A., Rull, R., Richardson, S., & Tu, X. M. (2019). Post hoc power analysis: is it an informative and meaningful analysis?. General psychiatry, 32(4), e100069.

Explicitly state in the abstract and discussion that findings are preliminary.

Thank you for the comment. Both the abstract and the discussion have been modified according to this comment to make it more explicit that we are introducing and discussing preliminary findings.

Consider using bootstrapped effect size CIs rather than relying solely on p-values.

Thank you for this suggestion. We now computed bootstrapped effect size CIs for each of our analyses.

The use of permutation ANOVA is appropriate for small samples, but: The manuscript does not specify whether permutations were within-subjects constrained (i.e., shuffling condition labels within each participant). If not, the test may be invalid for repeated measures. Clarify permutation scheme in Methods → Statistical analysis.

We thank the reviewer for the comment. Permutation tests were conducted using subject-constrained permutations. Specifically, in the coin: independence_test mentioned in the Method section, participant ID was included as a blocking factor, ensuring that condition labels were permuted only within each participant and preserving the repeated-measures structure of the data. We added this information in the statistical analysis section.

The authors do not discuss how the tiny sample affects statistical reliability and generalizability.

Thank you for noticing this. We reviewed the discussion and, where appropriate, we made the necessary changes.

2.2 Severe Inconsistency Between Behavioral & Modeling Results (Unresolved)

Behavioral data show vibration INCREASES Sample Entropy (SamEn) (Lines 293–305), but the neural model shows vibration DECREASES SamEn (Lines 306–320).

The authors merely mention this discrepancy but provide no plausible neurophysiological explanation (Lines 351–370). This renders the model’s validity questionable.

Thank you for pointing this out. We agree that this point needs a better clarification. First, It is important to note that the two entropy measures quantify fundamentally different dynamical systems and are not expected to vary in identical directions. We believe that the apparent discrepancy does not invalidate the model; rather, it represents one of its most compelling predictions. The model operates on the central representation of posture (i.e., the brainstem barycenter), rather than on the peripheral center of pressure (CoP). Achilles tendon’s vibration exerts distinct effects at the peripheral and central levels:

1. Peripheral level: tendon vibration introduces afferent sensory noise, thereby increasing the irregularity of the CoP signal and consequently increasing sample entropy.

2. Central level: in response to the degradation of proprioceptive input, the central nervous system (CNS) adopts a more rigid and stereotyped control strategy, resulting in more predictable brainstem dynamics and, consequently, a reduction in sample entropy.

This framework is fully consistent with the complexity theory literature on motor control (Stergiou & Decker, 2011), which claims that movement variability reflects the structure of the control process rather than simple noise. This apparent paradox arises because the CNS shifts toward more conservative control strategies under conditions of sensory uncertainty. Reference Pakniyat & Namazi, 2021, already cited in the manuscript, further supports this interpretation.

We added a dedicated paragraph (lines 448-466) to discuss this point.

Stergiou, N., & Decker, L. M. (2011). Human movement variability, nonlinear dynamics, and pathology: is there a connection?. Human movement science, 30(5), 869-888.

Pakniyat, N., & Namazi, H. (2021). Complexity-based analysis of the variations of brain and muscle reactions in walking and standing balance while receiving different perturbations. Frontiers in Human Neuroscience, 15, 749082.

The model replicates some behavioral trends (blurred vision → lower entropy) but shows opposite effects for vibration (model: decreased entropy; behavior: increased entropy). The authors interpret this as a “divergence between peripheral and central entropy,” but without direct neural recordings (EEG/fMRI), this remains speculation. Add a dedicated limitations paragraph acknowledging that model-behavior disagreement may indicate missing model components (e.g., spinal or muscle-level dynamics).

We thank the reviewer for this comment. We have modified the existing limitation section adding the suggested limitation related to the missing neural recordings. Limitations related to the missing descending pathways was already present in the section.

Show simulated CoP (not just brainstem barycenter) if possible, or explain why brainstem activity must correlate with CoP under all conditions.

Thank you. Explanations about brainstem activity correlation with CoP have been added in the Model simulation paragraph.

No behavioral effect of sound is found, but the model shows strong central effects. The conclusion that “dynamic auditory cues may have potential to impact CoP predictability” is speculative without: A control task where auditory cues are behaviorally relevant (e.g., spatial localization task). A more challenging balance task (e.g., Romberg, foam surface). Re-frame as hypothesis-generating, not evidence of a latent effect.

We thank the reviewer for the comment. The sentence has been reframed as requested.

“Predictability” is used to mean low Sample Entropy. Define explicitly in the introduction. “Rigid control strategy” (blurred vision) implies stiffness – this is a physiological claim not directly measured. Soften to “less flexible postural dynamics.” Abstract says “first time to our knowledge” – this is fine but verify that no prior neural mass model of postural multisensory integration exists (e.g., work by Kuo, van der Kooij, or Chiba).

We thank the reviewer for the comment. These issues have been addressed throughout the manuscript where needed. We rephrased the last sentence of the abstract to cautiously specify that no prior neural mass model investigated multisensory integration with respect to postural control predictability. None of the models cited by the reviewer employs Sample Entropy as an output metric, nor do they model the integration of auditory and postural sensory information.

2.3 Insufficient & Non-Replicable Methodological Details

Visual blur manipulation: No quantification (e.g., visual acuity, light transmittance) (Lines 103–110). Other researchers cannot replicate the setup.

Thank you for noticing this. We added more information about how our blurring technique affected normal vision, (lines 138-145) so that others can replicate this setup more closely now.

Vibration parameters: 180 Hz vibration intensity, duration, and contact pressure are not reported (Lines xxx) in the method section. Sample Entropy parameters (m=4, r=0.25): No sensitivity test is presented to justify these values for CoP data (Lines 160–170).

We added information about vibration parameters in the manuscript. About Sample Entropy parameters, we now report sensitivity analysis in the Supporting Information, Figure A2 and A3 in the Appendix A, and we refer to them in the manuscript.

Neural model parameters: Physiological justification for synaptic weights and noise scaling is missing (Lines 239–260).

We added a specific paragraph addressing this point (Lines 266-272 - Paragraph Model Simulation)

2.4 Poorly Designed & Uninformative Figures (Major Defect)

All figures are unpolished, low-quality, and missing standard statistical elements (Lines 288–320; Figures 1–4):

Figure 1: Simple schematic with no scale, no clear labeling of equipment, and low visual clarity.

Figure 3: Shows only individual data points without error bars (mean±SD/SEM); no statistical annotation; layout is messy and difficult to interpret.

Figure 4: Top panels: No error bars; significance asterisks are unclear.

Simulation panel: Y-axis scale is inconsistent; labels are vague; no units or statistical details.

General figure issues: No proper legends, no units, no definition of symbols; figures do not effectively support the results.

All figures must be completely redesigned and reconstructed to meet basic academic journal standards.

Thank you for these comments. All figures have now been redesigned to meet the academic journal standards as suggested.

2.5 Weak Neurophysiological Justification of the Model

The model excludes the vestibular system, which is essential for postural control (Lines 172–196). This is a major structural oversight.

Thank you for this comment. Although we strongly acknowledge that the vestibular system is integral to postural control, it was not manipulated across experimental conditions; all participants stood on a stable surface without galvanic vestibular stimulation or head perturbation. Therefore, vestibular input was modeled as a constant baseline drive to the cerebellar module, contributing to resting-state activity but not to condition-specific Sample Entropy differences. Its inclusion as a dynamic variable would have introduced a free parameter without empirical constraint from our data. This explanation is reported on lines 223-231 in the General model structure paragraph.

No model robustness test (parameter sensitivity analysis) is provided; results may be due to arbitrary parameter tuning.

We acknowledge that a formal parameter sensitivity analysis was not performed in the present study; this now constitutes a recognized limitation (page and lines), and future work will address it by adopting biologically validated neuro-musculoskeletal models of the spinal-peripheral effector stage (such as those developed by Elias and colleagues) that would also provide the physiological constraints needed to anchor parameter ranges more tightly. Nevertheless, as stated in paragraph General model structure, lines 209-210, parameters were tuned based on physiologically available information (see references [2,14,35,36] in the manuscript.

Elias LA, Watanabe RN, Kohn AF. Spinal Mechanisms May Provide a Combination of Intermittent and Continuous Control of Human Posture: Predictions from a Biologically Based Neuromusculoskeletal Model. PLoS Comput Biol 2014;10.

[2]Kandel ER, Schwartz JH, Jessell TM, Siegelbaum S, Hudspeth AJ, Mack S. Principles of neural science. 5th ed. New York: McGraw-Hill: 2000.

[14] Takakusaki K. Functional Neuroanatomy for Posture and Gait Control. J Mov Disord 2017;10:1–17. https://doi.org/10.14802/jmd.16062.

[35] Ramnani N. The primate cortico-cerebellar system: anatomy and function 2006;7:511–22. https://doi.org/10.1038/nrn1953.

[36] Horak FB, Macpherson JM. Postural orientation and equilibrium, 1996.

The model does not include descending motor pathways, so the link between central neural activity and CoP behavior is unsupported (Lines 401–410).

This has been clarified in the Discussion, specifically at the end of Proprioception modulation paragraph.

2.6 Superficial Discussion & Limited Novelty

The study is a simple combination of existing techniques (sample entropy + neural mass model) with no novel conceptual contribution.

Thank you for pointing this out. The novelty of the present work does not lie in the simple combination of sample entropy analysis with a neural mass model. Rather, it resides in the first application of a neurocomputational model to predict the sample entropy of central neural activity within a postural paradigm involving multisensory integration. None of the models previously mentioned by the reviewer (Kuo, van der Kooij, or Chiba) employs Sample Entropy as an output metric, nor do they model the integration of auditory and postural sensory information. Accordingly, the novelty of the proposed approach stems from the development of a computational framework capable of linking central neural dynamics, quantified through entropy-based measures, with multisensory mechanisms underlying postural control. We added a short sentence to clarify this novel aspect of the present work [Discussion paragraph].

The “auditory central modulation” finding is weakly supported and not placed in the context of recent multisensory integration literature.

Thank you. More literature about auditory central modulation has been added to the discussion.

Clinical implications are entirely missing; the translational value is not discussed.

Thank you. We added a paragraph about clinical implications after the main Discussion section.

3. Minor Concerns

Abbreviation errors: CoP, SamEn, SOT, AP, ML are not consistently defined at first use.

Writing quality: Poor grammar, awkward sentence structure, and disorganized logic; professional English editing is required.

We revised the whole manuscript accordingly.

Data transparency: Data availability states “will be available upon acceptance” but provides no repository link or data dictionary (Lines 426–430).

We added the link of the repository to our statement, which however will remain not accessible pending Institutional approval.

Limitations: The authors ignore key limitations (e.g., no dynamic postural tasks, no clinical population validation).

We improved the limitation section according to reviewer concerns.

Attachment Submitted filename: Response_to_Reviewers.pdf

# Peer review: Decision Letter 1

**DOI:** 10.1371/journal.pone.0355402.r003

**Authors:** Yih-Kuen Jan

22 Jul 2026

Disentangling Sensory Contributions to Postural Control Regulation Through Sample Entropy and Neural Modeling: a Preliminary Study

PONE-D-26-17082R1

Dear Dr. Montagnani,

We’re pleased to inform you that your manuscript has been judged scientifically suitable for publication and will be formally accepted for publication once it meets all outstanding technical requirements.

Within one week, you’ll receive an e-mail detailing the required amendments. When these have been addressed, you’ll receive a formal acceptance letter and your manuscript will be scheduled for publication.

An invoice will be generated when your article is formally accepted. Please note, if your institution has a publishing partnership with PLOS and your article meets the relevant criteria, all or part of your publication costs will be covered. Please make sure your user information is up-to-date by logging into Editorial Manager at Editorial Manager® and clicking the ‘Update My Information' link at the top of the page. For questions related to billing, please contact billing support.

If your institution or institutions have a press office, please notify them about your upcoming paper to help maximize its impact. If they’ll be preparing press materials, please inform our press team as soon as possible -- no later than 48 hours after receiving the formal acceptance. Your manuscript will remain under strict press embargo until 2 pm Eastern Time on the date of publication. For more information, please contact onepress@plos.org.

Kind regards,

Yih-Kuen Jan, PhD

Academic Editor

PLOS One

Additional Editor Comments (optional):

Reviewers' comments:

Reviewer's Responses to Questions

**Comments to the Author**

Reviewer #1: All comments have been addressed

**********

2. Is the manuscript technically sound, and do the data support the conclusions?

Reviewer #1: Yes

**********

3. Has the statistical analysis been performed appropriately and rigorously?

Reviewer #1: Yes

**********

4. Have the authors made all data underlying the findings in their manuscript fully available?

The PLOS Data policy

Reviewer #1: Yes

**********

5. Is the manuscript presented in an intelligible fashion and written in standard English?

Reviewer #1: Yes

**********

Reviewer #1: Dear Editor

Based on revision paper, i belived that the amendments made by the author are hereby approved.

**********

what does this mean?). If published, this will include your full peer review and any attached files.

If you choose “no”, your identity will remain anonymous but your review may still be made public.

**Do you want your identity to be public for this peer review?**  For information about this choice, including consent withdrawal, please see our Privacy Policy

Reviewer #1: **Yes:**  Amir Shams, Associate Professor, Sport Sciences Research Institute, Tehran, Iran

**********

# Editor report: Acceptance letter

**DOI:** 10.1371/journal.pone.0355402.r004

**Authors:** Yih-Kuen Jan

PONE-D-26-17082R1

PLOS One

Dear Dr. Montagnani,

I'm pleased to inform you that your manuscript has been deemed suitable for publication in PLOS One. Congratulations! Your manuscript is now being handed over to our production team.

At this stage, our production department will prepare your paper for publication. This includes ensuring the following:

* All references, tables, and figures are properly cited

* All relevant supporting information is included in the manuscript submission,

* There are no issues that prevent the paper from being properly typeset

You will receive further instructions from the production team, including instructions on how to review your proof when it is ready. Please keep in mind that we are working through a large volume of accepted articles, so please give us a few days to review your paper and let you know the next and final steps.

Lastly, if your institution or institutions have a press office, please let them know about your upcoming paper now to help maximize its impact. If they'll be preparing press materials, please inform our press team within the next 48 hours. Your manuscript will remain under strict press embargo until 2 pm Eastern Time on the date of publication. For more information, please contact onepress@plos.org.

You will receive an invoice from PLOS for your publication fee after your manuscript has reached the completed accept phase. If you receive an email requesting payment before acceptance or for any other service, this may be a phishing scheme. Learn how to identify phishing emails and protect your accounts at https://explore.plos.org/phishing.

If we can help with anything else, please email us at customercare@plos.org.

Thank you for submitting your work to PLOS ONE and supporting open access.

Kind regards,

PLOS ONE Editorial Office Staff

on behalf of

Dr. Yih-Kuen Jan

Academic Editor

PLOS One
